# The potential land requirements and related land use change emissions of solar energy

**DOI:** 10.1038/s41598-021-82042-5

**Published:** 2021-02-03

**Authors:** Dirk-Jan van de Ven, Iñigo Capellan-Peréz, Iñaki Arto, Ignacio Cazcarro, Carlos de Castro, Pralit Patel, Mikel Gonzalez-Eguino

**Affiliations:** 1 Basque Centre for Climate Change (BC3), Edificio Sede 1-1, Parque Científico de UPV/EHU, Barrio Sarriena S/N, 48940 Leioa, Spain; 2 Research Group on Energy, Economy and System Dynamics, Escuela de Arquitectura, University of Valladolid, Av Salamanca, 18, 47014 Valladolid, Spain; 3 Department of Economic Analysis, ARAID-Aragonese Agency for Research and Development, Agrifood Institute of Aragon (IA2), University of Zaragoza, Zaragoza, Spain; 4 Joint Global Change Research Institute, Pacific Northwest National Laboratory, 5825 University Research Court, Suite 3500, College Park, MD 20740 USA; 5 University of the Basque Country (UPV/EHU), Barrio Sarriena s/n, 48940 Leioa, Spain

**Keywords:** Environmental impact, Climate-change mitigation, Climate-change policy

## Abstract

Although the transition to renewable energies will intensify the global competition for land, the potential impacts driven by solar energy remain unexplored. In this work, the potential solar land requirements and related land use change emissions are computed for the EU, India, Japan and South Korea. A novel method is developed within an integrated assessment model which links socioeconomic, energy, land and climate systems. At 25–80% penetration in the electricity mix of those regions by 2050, we find that solar energy may occupy 0.5–5% of total land. The resulting land cover changes, including indirect effects, will likely cause a net release of carbon ranging from 0 to 50 gCO_2_/kWh, depending on the region, scale of expansion, solar technology efficiency and land management practices in solar parks. Hence, a coordinated planning and regulation of new solar energy infrastructures should be enforced to avoid a significant increase in their life cycle emissions through terrestrial carbon losses.

## Introduction

The technologies harnessing renewable energy sources are characterized by a power density several orders of magnitude lower than fossil fuels^1^. As a consequence, the transition to these sources of energy is expected to intensify the global competition for land^2–4^. For example, the sprawl of bioenergy has been already identified as the major driver of recent land use change (LUC) in developed regions^5,6^. Increasing land competition can cause various environmental impacts intensifying biodiversity loss, water use or indirect land use change (iLUC) emissions. The latter refers to emissions produced by using cropland for energy purposes and, therefore, indirectly increasing land competition elsewhere in the world to meet global food demand, potentially replacing land with high carbon stocks, such as natural forests^7–10^. For example, the literature estimates that the indirect land competition induced by liquid biofuels in developed regions leads to global land clearing and associated iLUC emissions higher than the emission savings achieved by replacing gasoline by these biofuels during 30 years^11–13^.

For sources of renewable energy other than bioenergy, land requirements and the associated environmental impacts remain understudied in the literature from a quantitative point of view^1,10^. In the case of solar energy, the land competition element is usually expected to be negligible due to its higher relative energy density compared to bioenergy and the possibility to integrate it in urban areas or non-productive land^7,14–16^, and as such is currently excluded from official statistical reporting and integrated assessment models (IAMs). However, recent studies based on satellite views of utility-scale solar energy (USSE) under operation, either in the form of photovoltaics (PV) or concentrated solar power (CSP), show that their land use efficiency (LUE) is up to six times lower than initial estimates^17–19^. Applying such observed LUEs accordingly reduces the potential contribution of solar on rooftop space^1,20,21^.

The installation of USSE on land is subject to a diversity of constraints: solar resource constraints, which are related to the solar irradiance in a certain area; geographical constraints such as the slope and the existing use of the land; and regulatory constraints, e.g. the protected status of the land, often related to ecosystem and wildlife preservation^21–27^. Therefore, where available, deserts and dry scrubland with high solar irradiance and which are generally not suitable for human activities, are used or planned to be used for solar energy^26–28^. However, beyond hard restrictions, other features such as the lack of road, electricity and water infrastructures, and the distance from human settlements complicate the large scale construction, operation and maintenance of solar power in these areas^22^. On top of that, spatial frictions might occur if land which is made available for solar energy by national or local governments is in reality a biodiversity hotspot^29,30^ or the home of human communities^31,32^. Recent developments show that USSE in densely populated countries is often installed on arable land that is used or potentially suitable for other productive uses such as agriculture or forestry^17,26,33,34^, intensifying land competition for the same reasons as the sprawl of bioenergy does. Furthermore, clearing currently vegetated land for USSE also has local impacts on biodiversity, carbon cycling and aestetics^25,30,35^.

The share of solar energy in global electricity scenarios that are largely or fully decarbonized by 2050 usually vary from about 20% to 60%^36,37^. For specific regions, these penetration levels can even range up to 90%^37^. Due to the potential relevance and relatively low power density of solar energy in a decarbonized future, and given that PV in urban areas will only be able to cover a share of the total demand^1,21^, this paper aims to quantify the potential land occupation of solar energy installed up to 2050, and the related direct and indirect impacts on carbon cycles, within a context of global climate action as proposed in the Paris Agreement. We concentrate on three regions with heterogeneous features where futures with a high solar energy penetration have been identified in the literature as likely to induce land competition: the European Union (EU), India and jointly Japan and South-Korea. Uncertainties in terms of future solar module efficiency improvements up to 2050 (20, 24, 28%) are taken into account, as well as solar land management options and their different associated impacts on local carbon cycles: depending on how the land below and around solar energy installations is managed, and on the land use prior to the conversion to solarland, land transformation for hosting USSE can cause a net release of carbon that was stored in soil and vegetation, or can lead to net carbon uptake^38^. See Section 2 of the Supplementary Material (SM) for an overview of the scenarios designed for this study.

## Results

A novel method has been specifically designed in this work which allows dynamically accounting for the land occupation of solar energy, depending on the geographical location and year of installation and based on real-world LUE observations^1,17^, within a state-of-the-art Integrated Assessment Model (IAM) that links energy, land, socioeconomic and climate systems (see “Methods” section) and that has also been applied in other studies to measure the terrestrial carbon leakage induced by bioenergy in a climate change mitigation context^9,39,40^. Through this model, a range of electricity mix penetration scenarios are simulated for solar energy technologies (and bioenergy for comparison). Based on the spatially defined LUE of solar energy, as well as the identified potential for solar energy in urban areas, deserts and dry scrublands, land use for solar energy competes with other land uses through the inherent relative profitability of each land use. The induced global land cover changes and related LUC emissions are then compared with scenarios where the same emission reduction targets in the electricity sector are achieved without solar and bioenergy, to isolate the additional land requirements, land cover impacts and related LUC emissions provoked by solar and bioenergy.

### Solar land occupation

**Figure 1:**
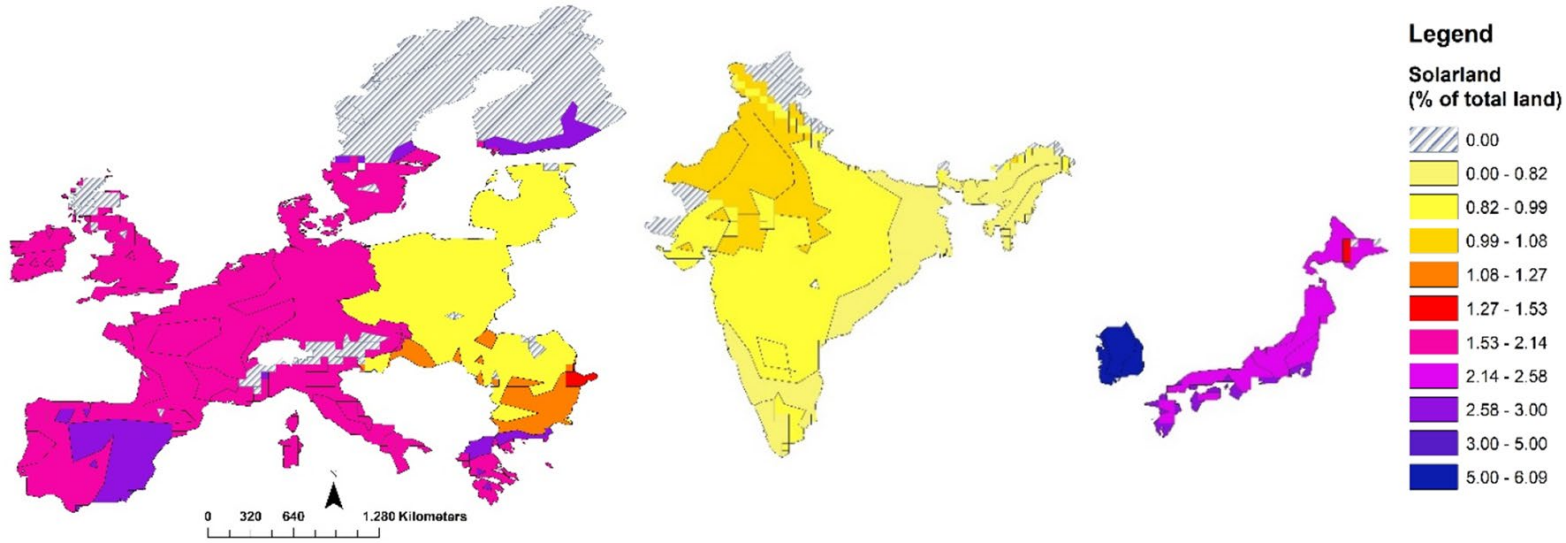
Geographical distribution of the share of total land occupied by solar energy within each region, by agro-ecological zone. See “Methods” section and Figure S1 of the SM for more information on the spatial resolution used in this study. Source: Authors´ own elaboration with the Arc GIS 10.5.1 Desktop (Esri) software.

Table 1 shows the obtained results for absolute and relative land requirements of solar energy, based on land that is (potentially) suitable for commercial production (i.e. crops, animal husbandry, and forestry, so excluding the use of rooftops deserts and dry scrublands), for the simulated scenarios at penetration rates ranging from 26 to 79% of the electricity mix, and for the range of future solar PV module efficiencies. Due to the lower irradiance and higher latitude of Europe, absolute land use of per unit of solar output is almost twice as high as in Japan and South-Korea and three times higher as in India (see Fig S6 in the SM). This ratio increases with higher penetration rates, due to the satiation of the potential to generate solar energy on rooftops (see also Figure S12 in the SM) in combination with the decreasing marginal returns for land-based solar energy. With solar energy accounting for 25 to 80% of the electricity mix, land occupation by USSE is projected to be significant, ranging from 0.5 to 2.8% of total territory in the EU, 0.3 to 1.4% in India, and 1.2 to 5.2% in Japan and South-Korea. This occupation is unequally spread within each of the regions, as areas that are relatively attractive for solar energy are prioritized in each region, such as southern Europe, north western India, and southern Japan and South-Korea (see Fig. 1).

**Table 1:** Land occupation characteristics at different solar penetration levels by 2050.

Focus region	Solar penetration by 2050^a^	Rooftop PV generation share of solar (2050)	Occupation of land suitable for commercial purposes (^b^) by 2050		Relative solar land occupation by 2050^c^			Occupation of land suitable for commercial purposes (^b^)
	% of total electricity (PWh in 2050)	% of solar penetration by 2050	Solar energy	Bioenergy (% within region)	% of total land area	Compared to urban area in 2010 (%)	Compared to crop area in 2050 (%)	km^2^ per TWh solar (average 2020–2050)
			1000 km^2^					
European Union	26 (1.19)	24.3–23.0	21–28	366 (45)	0.5–0.7	20–27	1.9–2.5	19.4–24.2
	53 (2.54)	12.3–11.6	53–69	614 (38)	1.3–1.7	50–66	4.8–6.3	22.1–28.0
	79 (3.87)	8.1–7.6	85–111	969 (32)	2.1–2.8	81–106	7.7–10	23.5–29.7
India	30 (1.8)	10.6–9.9	10–14	596 (16)	0.3–0.5	46–62	0.6–0.9	6.4–8.2
	54 (3.29)	5.9–5.5	20–26	1051 (12)	0.7–0.9	88–118	1.2–1.6	6.5–8.5
	78 (4.88)	3.6–3.3	30–41	1516 (10)	1.0–1.4	137–182	1.9–2.5	6.9–8.8
Japan and South-Korea	28 (0.5)	25.0–22.1	5–6	185 (17)	1.2–1.6	36–48	8.3–11	12.9–15.6
	46 (0.8 PWh)	15.6–13.8	9–12	279 (13)	2.3–3	68–89	16–21	13.3–16.4
	74 (1.3 PWh)	9.0–8.1	16–21	429 (10)	4–5.2	120–157	29–39	13.9–17.1

Ranges show results for with different future solar module efficiencies with left values within each solar-related column representing 28% efficiency and right values 20%. Results for bioenergy scenarios included for comparison.

^a^These are realized penetration levels. See Section 1b in the SM for more information.

^b^Land suitable for commercial purposes does not include the use of rooftop space, deserts or dry scrublands that are not suitable for crop, pasture or forest cultivation. Deserts and dry scrublands in India host about 11.5–12% of solar energy throughout all penetration scenarios of solar energy in India (see Figure S8 in the SM). See the “Methods” section and Section 2b in the SM for modelling details with respect to location choices.

^c^These columns compare the “Land suitable for commercial purposes by 2050” to total land, urban land (in 2010; future urban expansion is not modelled) and total crop area (abstracted from the same modelled scenarios).

The future land requirements of solar energy obtained for each scenario and region can be put in perspective compared, for example, to the current level of built-up area and agricultural cropland. In the three regions, a large part of the total built-up area (urban and solar land) will consist of solar PV panels or CSP heliostats by 2050 if at least half of the produced electricity comes from solar power. Land for solar would amount to over 50% of the current EU urban land, over 85% for India, and over 75% in Japan and South-Korea. From a different perspective, a significant part of the sunlight captured for commercial use would be used for electricity generation instead of growing crops, especially in Japan and South-Korea (29–39%) and the EU (8–10%). The relative projected land area dedicated to either crops or solar energy strongly differs within each region, with potential local ecosystem and landscape implications (see Figure S16 in the SM).

### Land cover changes

**Figure 2:**
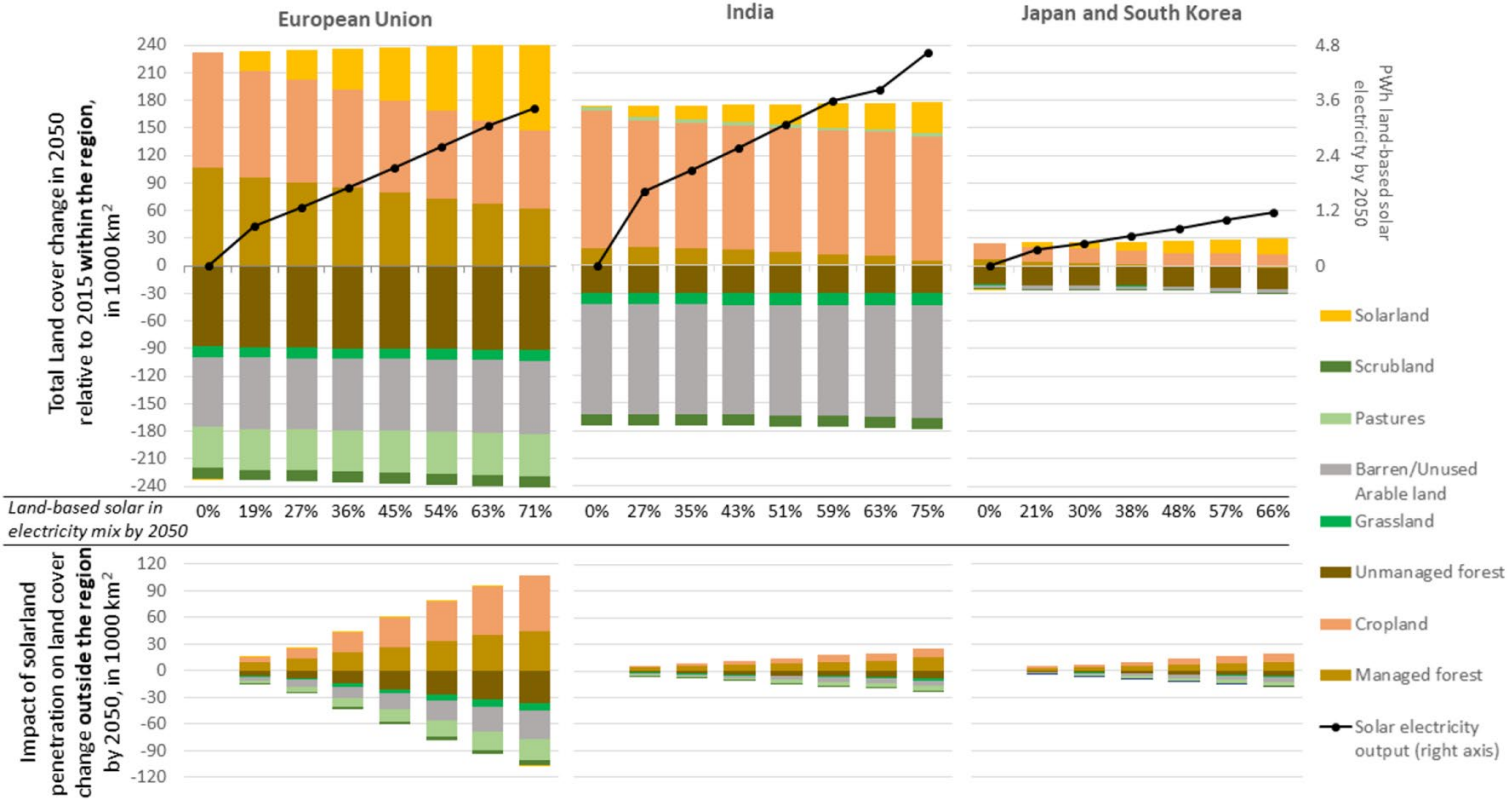
Global land-cover changes by 2050 due to solar expansion, for a range of solar energy penetration levels and for an average efficiency of installed solar modules of 24% by 2050. The upper graphs shows total land cover changes by 2050 relative to 2015 within each region and the lower side shows the land cover changes in the rest of the world (leaking), indirectly driven by the penetration of solarland within the region. Positive land cover changes refer to increases and negative to land cover loss. See Section 3b in the SM for aggregated global land cover changes. Note that land cover changes do not correspond with land use changes: this figure compares total land cover in different scenarios of land-based solar energy penetration, but does not show which specific types of land convert to solarland (or any other type of land). Note that these land cover changes are based on simulated land use decisions driven by economic optimisation. See “Methods” section for more details.

Solar energy infrastructure currently occupies a negligible amount of land globally. Our results show that this changes in scenarios with a high share of solar energy in the future electricity mix. Figure 2 shows the obtained land cover changes related to increasing solar energy (see Table 1), within each of the three regions (upper part of the figure), and indirectly driven land cover changes outside of these regions in the rest of the world (lower part). Based on assumptions on economic and suitability constraints (see Section 1c in SM), solar energy expansion in the three regions is found to predominantly replace (or avoid future land conversion to) land used for commercial purposes, such as cropland or commercial forest (e.g. for timber products or biomass). Instead, solar energy penetration is not found to significantly affect the cover of unmanaged land in each of the three regions. However, the displacement of commercial land within each of the three focus regions would incentivise the use of currently unused arable land in other regions, while also boosting the commercialisation of unmanaged land, indirectly leading to the loss of natural land cover. The magnitude of this indirect land cover impact depends on the crop and forestry productivity in regions where solar energy penetration takes place: relatively high crop productivities in the EU, Japan and South-Korea mean that the displacement of cropland from these regions to regions with lower crop productivities would indirectly increase global cropland cover, amplifying the impact of solar energy expansion in these regions on global land competition by up to 22%. This effect is lower at lower solar energy penetration levels (even negative in the EU), as solar energy is projected to displace the most marginal cropland first. In India, where current and projected crop productivities are below the global average, the impact of solar expansion on global land competition is less significant.

Figure 2 shows that, either directly or indirectly, expansion in solar energy predominantly reduces non-commercial land cover on a global scale: for every 100 hectares of solarland in the EU, we find that, depending on the solar penetration level, 31 to 43 hectares of unmanaged forest may be cleared throughout all the world. The same amount of solarland in India would clear 27 to 30 hectares of unmanaged forest, and for Japan and South-Korea, the ratio is 49 to 54 hectares.

### Impact on terrestrial carbon stocks

The land cover changes in Fig. 2 imply that solar expansion leads to LUC emissions, such as iLUC emissions related to increasing global land competition, emissions related to vegetation loss if forest and scrubland makes place for solarland (either directly through deforestation or indirectly by avoiding future afforestation), and carbon release from soil and vegetation directly below the installed panels, where sunlight is much reduced^35^. However, an important part of the emission balance is related to the land management regime applied in solarland. If all vegetation is cleared and avoided to regrow through the application of herbicides, which is a common practice in various countries^41^, LUC emissions from solar expansion are further amplified. In contrast, if arable land plots are converted to solar parks whose surface is managed as pastures, there will be a net carbon sequestration in vegetation and soil in the decades following upon the conversion (apart from the land directly below the panels, where photosynthesis is largely blocked)^35^, offsetting some or all of the inevitable LUC emissions caused by land competition. In reality, the application of a particular land management practice depends on a diversity of local factors (policies, climate, etc.). See “Methods” section for a detailed explanation of each land management regime.

**Figure 3:**
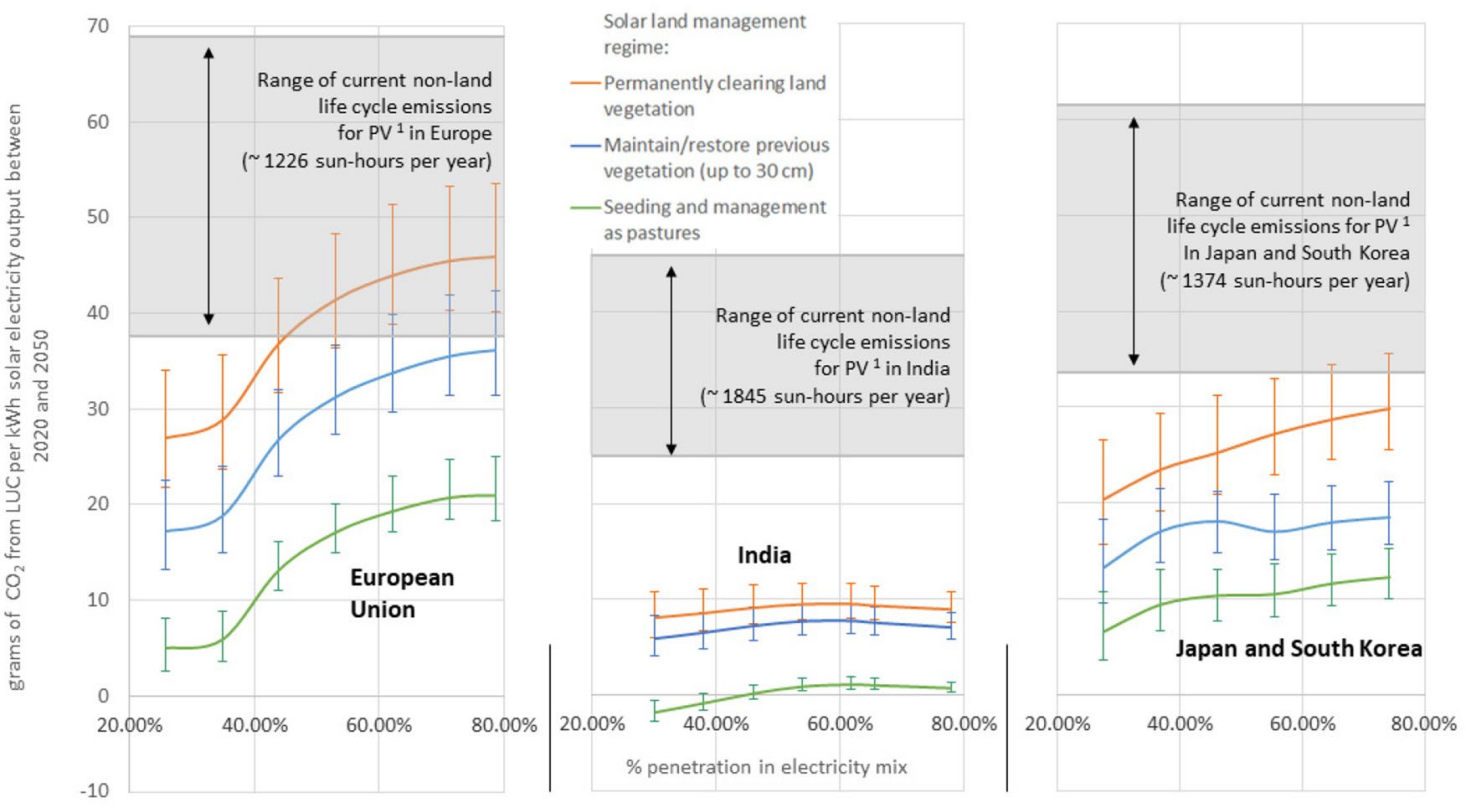
Land use change emissions related to land occupation per kWh of solar energy from 2020 to 2050, for the three solarland management regimes applied (see “Methods” section for more details), and relative to other life cycle emissions of PV systems (depend on location of installation) and emissions from natural gas fired electricity (independent of location). Uncertainty bounds reflect solar module efficiency scenarios (reaching average efficiencies of 20, 24 and 28% for modules installed in 2050; see Section 2c in SM). ^1^ Non-land life cycle emissions of PV are based on a range of PV technologies, including mono and multicrystalline silicon (higher range), thin-film CdTe (lower range), CIS and a-Si systems as calculated in Liu & van den Bergh (2020)^42^, and based on an average global carbon intensity of electricity (0.48 kg CO_2_/kWh). The range is calculated by dividing the regionally weighted solar electricity output per m^2^ as used in this study, by CO_2_ emissions per m^2^ panel surface.

Figure 3 and Table 2 show the obtained LUC emissions per unit of solar energy installed from 2020 to 2050 associated to the different simulated solar penetration and module efficiency scenarios, and for different management regimes of the land in solar parks. They show that solar expansion scenarios until 2050 will most likely lead to net LUC emissions, although there can be a net carbon sequestration in India when managing the land in solar parks as pastures. The sequestration effect is amplified if delayed post-2050 impacts on local carbon cycles are taken into account (see Table 2). In the absence of land management practices specifically aiming at carbon sequestration, land cover change due to the expansion of solar energy in the EU would cause 13 to 53 g of CO_2_ per produced kilowatt-hour (kWh) of electricity, about 4 to 16% of the CO_2_ emissions from natural gas fired electricity. Solar energy in India involves significantly less land cover change per unit of output (see Fig. 2), and estimated LUC emissions per kWh are below 12 g of CO_2_ for all scenarios. In Japan and South-Korea, LUC emissions related to the expansion of solar energy are 11 to 35 g of CO_2_ per kWh. When using relatively efficient PV technologies such as monocrystalline and multicrystalline silicon (made from a single crystal of silicon and from many silicon fragments melted together, respectively) (lower range of estimated LUC emissions, higher range of non-land life cycle emissions), our results show that LUC emissions are comparable to about 10 to 50% the current non-land life cycle emissions for such technologies. Instead, when using less space-efficient but more resource-efficient PV technologies such as thin-film Cadmium telluride (CdTe) made by depositing one or more thin layers of photovoltaic material on a glass, plastic or metal substrate (higher range of LUC emissions, lower range of non-land life cycle emissions), we estimate LUC emissions in the range of 50 to 150% of the non-land life cycle emissions. If solarland is seeded with herbs and managed as pasture, net LUC emissions drop by more than 50% in most cases.

**Table 2:** Land use change emissions and payback periods for solar penetration and solarland management scenarios.

	Solar penetration level	Land management scenario in solarland^a^	Direct and indirect land use change (LUC) emissions due to solar energy^b^			LUC emissions per occupied m^2c^		LUC CO_2_ payback period when replacing gas-fired electricity ^d^	
	% and PWh in 2050 elect. mix		Within region	Outside region	Total	Solar energy	Bio-energy	Solar energy	Bio-energy
			Grams of CO_2_ per kWh of solar electricity output between 2020 and 2050 (average)			kg CO_2_ (2020–2100) per m^2^ of dedicated land in 2050		Months	
**European Union**	26% (1.19 PWh)	CLEAR	28.1 to 38.6	− 6.4 to − 4.6	21.8 to 34.0	4.4 to 5.2	3	4.7 to 7.1	46.9
		MAINT	19.7 to 27.1		13.3 to 22.5	3.1 to 3.7		3.2 to 5.2	
		SEED	9.0 to 12.7		5.0 to 8.1	1.1 to 1.7		1.2 to 2.4	
	53% (2.54 PWh)	CLEAR	33.0 to 43.5	3.3 to 4.7	36.3 to 48.3	6.7 to 6.8	3.1	6.2 to 8.2	49.2
		MAINT	24.0 to 30.9		27.3 to 36.6	5.3 to 5.4		4.8 to 6.5	
		SEED	11.7to 15.4		15.0 to 20.1	~ 3.1		2.9 to 3.8	
	79% (3.87 PWh)	CLEAR	34.6 to 46.7	5.6 to 6.9	40.2 to 53.6	7.2 to 7.3	3	6.4 to 8.3	49.3
		MAINT	25.8 to 35.5		31.4 to 42.4	~ 5.9		5.2 to 6.7	
		SEED	12.7 to 18.1		18.3 to 24.9	3.5 to 3.6		3.2 to 4.0	
**India**	30% (1.8 PWh)	CLEAR	10.0 to 12.5	− 4.0 to − 1.6	6.0 to 10.8	1.3 to 2.4	2.3	0.3 to 0.8	41.7
		MAINT	8.1 to 10.0		4.1 to 8.4	0.6 to 1.7		0.2 to 0.6	
		SEED	1.1 to 1.3		− 2.7 to − 0.6	− 5.2 to − 6.2		− 1.5 to − 1.7	
	54% (3.29 PWh)	CLEAR	10.8 to 13.0	− 2.8 to − 1.3	8.0 to 11.7	2.5 to 3.1	2.3	0.6 to 1.0	43.2
		MAINT	9.1 to 10.8		6.3 to 9.5	1.9 to 2.4		0.5 to 0.8	
		SEED	3.0 to 3.2		0.4 to 1.7	− 4.5 to − 5.0		− 1.2 to − 1.5	
	78% (4.88 PWh)	CLEAR	9.7 to 11.7	− 2.1 to − 0.9	7.6 to 10.8	2.9 to 3.2	2.4	0.7 to 1.1	43.9
		MAINT	8.0 to 9.5		5.9 to 8.6	2.1 to 2.5		0.5 to 0.8	
		SEED	2.3 to 2.5		0.4 to 1.4	− 4.4 to − 4.8		− 1.2 to − 1.5	
**Japan and South-Korea**	28% (0.5 PWh)	CLEAR	18.9 to 25.8	− 3.2 to 0.8	15.7 to 26.6	4.8 to 6.8	2.7	2.9 to 5.2	47.7
		MAINT	12.8 to 17.5		9.6 to 18.2	2.2 to 4.1		1.4 to 3.1	
		SEED	6.8 to 10.0		3.6 to 10.7	− 0.3 to − 2.2		− 0.2 to − 1.3	
	46% (0.8 PWh)	CLEAR	23.0 to 30.6	− 2.1 to 0.6	20.9 to 31.1	6.1 to 7.2	2.6	3.3 to 5.0	47.3
		MAINT	16.9 to 20.5		14.8 to 21.1	3.9 to 4.3		2.1 to 2.9	
		SEED	9.9 to 12.5		7.7 to 13.1	− 0.2 to − 1.2		− 0.2 to − 0.7	
	74% (1.3 PWh)	CLEAR	25.6 to 33.3	0.0 to 2.3	25.6 to 35.6	7.1 to 7.7	2.7	3.6 to 5.0	48.9
		MAINT	15.6 to 20.0		15.6 to 22.2	3.5 to 3.9		1.8 to 2.6	
		SEED	10.0 to 12.9		10.0 to 15.2	− 0.5 to 0.0		− 0.3 to 0.0	

Ranges show results for with different future solar module efficiencies with left values within each solar-related column representing 28% efficiency and right values 20%. Results for bioenergy scenarios included for comparison.

^a^CLEAR: permanently clearing land vegetation; MAINT: Maintain/restore previous vegetation (up to 30 cm); SEED: Seeding and management as pastures. See “Methods” section for a detailed description of these land management scenarios.

^b^Dividing all LUC emissions from 2020 to 2050 to the total amount of generated electricity (including non-land-based sources, such as solar rooftops, unproductive land or waste-to-energy plants for bioenergy).

^c^Dividing all LUC emissions from 2020, including delayed carbon release or sequestration until 2100, by the total land area dedicated to solar and bioenergy by 2050 (maximum point). Negative values indicate net carbon sequestration for every m^2^ of solarland.

^d^Calculated assuming a thermal efficiency of 50% for natural gas power plants. Only taking account direct combustion emissions.

Table 2 also shows the obtained emissions per m^2^ of land occupation by solar energy, which reflect the value of the used land in terms of its potential to sequester carbon: either directly by its capacity to sequester carbon in soil and vegetation, or indirectly by its agricultural productivity which, if being displaced by solarland, will lead to conversion of non-commercial land to agricultural land elsewhere. Since in our simulations land for USSE predominantly replaces commercial land growing crops or timber products within each region (see Fig. 2), solar energy expansion displaces commercial timber production to other regions, indirectly increasing carbon sequestration outside the region by incentivising currently degraded forest or other arable land to be commercialised for timber production. At higher solar penetration rates however, increasing land pressure causes more natural forests to be used for timber or crop production, leading to higher land use change emissions outside the region. This effect is best visible for solar penetration scenarios in the EU, due to the high absolute amount of land use.

### Solar energy versus bioenergy

IAMs which link energy, economy, land and climate modules tend to rely strongly on the cultivation of dedicated bioenergy crops (such as switchgrass and miscanthus) in global climate change mitigation scenarios^43^. As the land use impacts of bioenergy have been extensively analysed in other studies, using the same model^9,39^, we proceed to compare the land occupation and related LUC emissions of electricity production from solar energy and bioenergy, with the purpose of improving the comparability of the obtained results.

Table 1 shows that land requirements for reaching certain levels of electricity penetration with solar energy are about a magnitude lower than land requirements to meet those same levels with bioenergy. Comparing the additional global LUC emissions until 2100 as a result of reaching certain shares of bioenergy in the electricity mix of 2050 in the regions in this study, we observe from Table 2 that emissions per dedicated m^2^ are in many cases lower than for solar energy at the same penetration level in the electricity mix. However, the energy density of solar energy is a magnitude higher than that of bioenergy. By comparing the total LUC emissions from one unit of solar and bioenergy to the “avoided” periodical combustion emissions from natural gas fired electricity, we calculate the “CO_2_ payback period” of these renewable alternatives for electricity production, which is a common method to compare LUC emission impacts of different types of bioenergy^13,44^. Table 2 shows that the payback period of bioenergy is significantly higher (~ 4 years) than that of solar energy (< 8 months), as the higher land requirements for bioenergy more than offset the lower emissions per m^2^ found in most cases. However, since the physical characteristics of bioenergy allow for trade over large distances, comparable to fossil fuels and in contrast to electricity from solar energy, only a limited part of the land requirements and related LUC emissions driven by bioenergy expansion is projected to be within the EU, India, Japan and South-Korea. Note that these results do only focus at solar and bioenergy based in land with potential commercial use. Solar energy in urban areas, deserts and dry scrublands, as well as bioenergy from waste or agricultural and forestry residue, are assumed not to contribute to LUC emissions nor carbon sequestration.

## Discussion

By representing the land requirements of solar energy within an IAM that integrates energy, land, socioeconomic and climate systems, we were able to, for the first time in the literature to our knowledge, estimate the land cover impacts and related LUC emissions of solar energy within climate change mitigation scenarios up to 2050. The obtained results represent a contribution to the novel field of research which analyses the environmental impacts of significantly up scaling renewables other than biomass^45,46^.

A combination of technical and geopolitical reasons complicates the installation of solar energy far from consumption points. Therefore, a high share of solar generation in the energy mix in relatively densely populated regions with high per capita energy demands can require a significant share of domestic land, comparable to the current built-up area in these regions. The most relevant factors influencing the land use per unit of solar energy are solar irradiation, latitude, and future solar module efficiencies. At the domestic level, solar energy is found to predominantly compete for land with cropland and managed forests, while on a global scale, 27 to 54% of the land required for solar energy is found to indirectly displace unmanaged forests, predominantly outside the region where the solar energy is consumed. Note that this iLUC has been documented to happen for biofuels^11–13^, although the strength of this effect is not comparable for solar energy given that the power density of solar is much higher than that of biofuels. Still, we do find a non-negligible effect in this study. The obtained land cover change imply environmental consequences such as greenhouse gas emissions and biodiversity loss^47^. However, the impact of USSE on local environmental impacts depends strongly on how this new solarland will be managed. If all previous vegetation is permanently cleared, the total (direct and indirect) LUC emissions related to the expansion of solar energy from 2020 to 2050 correspond to 5 to 16% of emissions from natural gas combustion for power generation in developed regions such as the EU, Japan and South-Korea, and about 2.5–3.5% in India, where conditions for solar energy are more favourable and crop yields are lower, implying less indirect emissions. However, if solarland is seeded with herbs and managed as pastures, total LUC emissions per kWh of electricity in the studied period are 3 to 5 times lower, and could even be negative (i.e., becoming net sources of carbon sequestration) in India, Japan and South-Korea, if long-term effects (post 2050) are taken into account.

Numerous Life Cycle Assessments (LCA) have been performed for solar energy, estimating the life cycle emissions of solar energy systems depending on many factors, such as the year and location of construction, solar module efficiency, mounting system, location of input production, among others^42,48^. Comparing the non-land life cycle emissions from LCAs to the LUC emissions estimated in this study, we can conclude that LUC emissions (which are normally not included in LCAs) increase total life cycle emissions of new USSE projects by 10 to 150% in the absence of land management practices focused on sequestering carbon in solarland, depending mainly on the region where the infrastructure is installed and the type of technology used. While this is a notable increase in life cycle emissions, it is also important to consider that LUC emissions will not repeat if a solar plant is renewed or upgraded after the initial construction phase, and therefore average LUC emissions of solar energy will be lower in the future. Also, this terrestrial part of solar energy life cycle emissions could be avoided by applying land management practices focused on carbon sequestration in solarland.

Using an existing IAM to study the potential land impacts of solar energy expansion, we were bound to the limitations of this model. One of these was the division of land zones in the model (corresponding to Agro-Ecological zones, see “Methods” section), which determine the boundaries of the geographical competition to host solar energy within each region. This pre-defined distribution was originally designed to capture variations in crop yields, and is not ideal for defining the geographical diversity of solar energy “yields” within a region. Although a general good correspondence is found, there are also exceptions (see Figure S6 in the SM). This limitation could be dampened in future work by using/developing a land cover layer that matches better with geographical differences in solar irradiation and latitude. We were also not able to account for the suitability of land for solar energy limited by the slope or the protection of the land^24^. Therefore, we implicitly assumed that those hectares that are converted to solarland in our scenarios are indeed suitable for hosting solar energy. In contrast, some land is suitable for solar energy and not for commercial crops or forests, such as dry scrubland and deserts, which are by default excluded from land competition in the applied model. The inclusion of a solar potential on identified “wastelands” in India (see “Methods” section) should have largely circumvented this inherent limitation in the applied method. To extend the analysis performed in this study to other regions, it is important to have a well-quantified potential for solar energy in areas that are not suitable to host other commercial land uses such as agriculture and forestry. Finally, we have not taken into account the potential to integrate solar systems in agricultural land (agrivoltaic systems), a technique that is currently in an early stage of research and development and of which the large-scale performance is still uncertain^49^.

To date, land use for solar energy is negligible compared to other human land uses. However, the obtained results show that in future scenarios, with a largely decarbonized electricity system, high penetration rates of solar energy will require significant amounts of land to be occupied by solar power plants. Further work applying ecological tools should be focused towards investigating the implications of these additional land occupation levels -including the additional transmission power lines- in terms of habitat fragmentation and ecosystem disturbance. Siting policies for USSE should avoid adverse land impacts and limit land competition, for example by excluding high yield cropland as already performed in some countries^50^, maximising the use of urban areas and degraded arable land^22^, or by seeding solarland with herbs and managing these lands as common pastures (e.g. by allowing extensive animal grazing), converting solarland to a net source of carbon sequestration^35^. Such techno-ecological synergies are crucial for minimising the unintended consequences of solar expansion^38^, such as the potential impacts on land cover change and LUC emissions as measured in this study. The results in this study also indicate that minimum efficiency standards for solar modules help to reduce solar land requirements and limit land competition, although there might be a trade-off with non-land life cycle impacts, which tend to be higher for high-efficiency solar modules. Finally, the inclusion of this new type of land use in integrated energy-land-climate models, as has been done in this paper, will be useful to capture a larger range of implications of specific energy transition scenarios.

## Methods

The Global Change Assessment Model (GCAM), version 4.3, has been used as a base for this study^51^. GCAM is a dynamic-recursive model with technology-rich representations of the economy, energy sector and land use linked to a climate model that can be used to explore climate change mitigation policies including carbon taxes, carbon trading, regulations and accelerated deployment of energy technologies. See Section 1a of the SM for a wider description of the model. The background scenario for the model exercises in this study consist of the “Middle of the Road” Shared Socioeconomic Pathway (SSP 2)^52^ with global CO_2_ reduction targets as defined by the Nationally Determined Contributions (NDCs) with increased ambitions after 2030^53^.

In order to identify the effects that solar energy and bioenergy pathways have on land use and land use change emissions, three pathways have been modelled achieving a defined penetration level in the electricity mix from 2020 to 2050, using different electricity generation technologies (see Section 2b in the SM on how the different penetration levels have been modelled):

- Solar energy pathway (S): land-based PV, rooftop-based PV, CSP
- Bioenergy pathway (B): Conventional biomass and biomass gasification (with and without Carbon Capture and Storage), Biomass-driven Combined Heat and Power.
- Non-land-occupying pathway (NL): wind, geothermal, rooftop-based PV (and nuclear in scenarios where penetration level cannot be reached with the first 3 technologies together)

The land occupation of solar and bioenergy (Figs. 1 and 2, Table 1) is identified using Eq.  (1), land use change emissions per unit of output from 2020 to 2050 (for Fig. 3 and Table 2) from 2020 to 2050 have been calculated using Eq. (2), and the CO_2_ payback period (Table 2) has been calculated using Eq. (3). In these equations, the subscript *r* defines the region, *p* the electricity penetration level, *i* the technologies included in either the solar- or bioenergy pathway, *NL* defines non-land-occupying energy technologies and *i(l)* represents land-competing solar- or bioenergy, so not taking into account solar energy based on rooftops, deserts or dry scrublands or bioenergy from waste or agricultural residues. The parameter *a* defines the CO_2_ emission factor per unit of electricity output of the alternative thermal generation technology (i.e. natural gas). Scenarios are run until 2050, but delayed effects on carbon release or sequestration in vegetation and soils can be abstracted until 2100. The impact from land management regimes have been calculated through off-model calculations, as such regimes are assumed not to affect the allocation procedure of new solar energy. See Section 2d of the SM for more details.

1 $${Land\, occupation}_{i,p,r}={land\, for \,i}_{i,p,r}-{land \,for\, i}_{NL,p,r}$$

2 $${LUC\, per \,output \,unit}_{i,p,r}= \frac{\sum_{p,r}^{2020\, to\, 2050}{(LUC}_{i}-{LUC}_{NL})}{\sum_{p,r}^{2020\, to\, 2050}{(output\, i}_{i}-{output\, i}_{NL})}$$

3 $${{CO}_{2} \,payback \,period}_{i\left(l\right),p,r,a}= \frac{\sum_{p,r}^{2020 \,to\, 2100}({LUC}_{i}-{LUC}_{NL})}{{output}_{i(l)}^{2050=max}* a}$$

### Land competition in GCAM

Land use and agricultural output in GCAM version 4.3 are calibrated for pre-defined Agro-Ecological Zones (AEZs), which sub-divide geo-political regions in 18 different types of land regions, based on differences in climate zones (tropical, temperate, boreal) and the length of growing periods for crops^54^. See Figure S1 in the SM for an overview of the AEZs within the three focus regions of this study.

Land use in GCAM has been divided in different nodes that affect the level of competition between different uses (see Figure S3 in SM). Those land-use categories (e.g. corn, wheat, bioenergy) belonging to the same node (crops in this example) are assumed to compete more directly with each other than with those land-uses in other nodes (e.g. forest or pasture). For each land use, assumptions on carbon stocks in the vegetation and the soil are made (see Table S1 in SM). A change in land cover either leads to positive or negative LUC emissions, driven by the difference in the assumed carbon stocks (in vegetation and soil) between the original and the new land use. Based on the profitability of each land use, which depends on assumed yields, production costs and commodity prices, land owners choose between different land uses to maximise profit. Such land use decisions are based on the logit model of sharing, taking into account the heterogeneity of local circumstances within each AEZ, and avoiding extreme “winner-takes-it-all” outcomes^55^. See Section 1a in the SM for more details, and see Wise et al.^56^ for a detailed explanation on the approach and design of the land module in GCAM.

### Solar land-use module

An additional module has been developed for the GCAM model to link the consumption of solar energy with land use, competing with other commercial (crops, timber and intensive pastures) and non-commercial (natural forest, grassland, scrubland) land uses. Specifically, the solarland category is included in the “Crops” land node (Figure S3 in SM), which means that demand for solarland will primarily compete with used, degraded and potential cropland (including chemically fertilised meadows). Indirectly, solarland also competes with other land uses such as forest, grass- and scrubland. This structure is based on observed tendencies for solar siting in Europe, India, Japan and South-Korea (see Table S2 in SM), showing that mainly arable land is used for current USSE projects, and supported by academic literature^17,33,34,57,58^ and solar industry reports^59,60^. Also, the optimal microclimate for solar energy production (based on insolation, air temperature, wind speed and humidity) is found over land that is currently used as cropland^61^, supporting the assumption that future investors will have a slight preference for cropland (in use or fallow) for the allocation of solar energy projects, among other factors such as flatness and connectivity in terms of roads and electricity grids^22^. Nevertheless, an important driver for land use decisions in the model is land profitability: even if land covered by crop cultivation is perceived as the most suitable by investors in solar energy, high observed or potential profitability of crop cultivation on such land could force investors to focus on other land types.

To define the value of land for hosting solar energy, a yield in terms of energy output per unit of land has been defined for every AEZ. Equation (4) defines this yield for each AEZ, which depends on average solar irradiation (*I*) per AEZ, average efficiency of solar power plants (*f*_*1*_) at the year of installation (*t*), the averaged performance ratio over the life cycle of the solar power plant (*f*_*2*_) and the land occupation ratio (*f*_*3*_)^1,17^. To estimate *I* per AEZ, we overlapped the solar irradiance annual average data^62^ (tilt radiation, i.e. the position where the tilt coincides with the latitude, which is the optimal position of PV panels to take advantage of the solar resource at each location) with each AEZ and geopolitical region in GCAM 4.3 using a GIS tool. The land occupation ratio, defined by Eq. (5), depends on the packing factor (*PF*) and the Generator-to-system area (*GSR*). PF is the ratio between the PV panels or heliostats and the ground area required for arrays’ installation including separation to avoid excessive self-shading, while GSR represents the share of the full area enclosed by the site boundary of the power plant which is covered by the PV panels and heliostats including the separation between them. Hence, with relation to the PF, the GSR accounts for the additional space required to host physical infrastructure such as access roads, substations service buildings, and other infrastructure, as well as land not being able to be directly used due to orography and unevenness of the plot preventing the optimization of the layout of the solar arrays. GSR is dependent on the size and shape of the terrain and plots and should be analysed on a case by case basis. GSR ranges of 0.7–0.85 have been reported^63^ although larger plants tend to have lower GSR due to more difficult use-optimization of land plots at large scale productions, hence here we take a GSR of 0.7 assuming that the deployment of scale of solar power plants on land will likely be based on larger-size plants due to the incentives of economics of scale^17,19^. The packing factor again depends on the average latitude of each AEZ and is defined by Eq. (6): the further from the equator, the more space is needed between the different panels or heliostats to avoid self-shading, so the lower the packing factor. The theoretical equation of PF dependent on the sun elevation, the sun azimuth and the tilt angle, which can be simplified assuming that tilt coincides with the latitude (β = ∅) and taking the conservative shading criterion of avoiding shading only at noon^63^. This formula is only valid for latitudes < 66.5° (to ensure PF > 0), but in this study we constrained solar deployment in high latitudes areas since low solar irradiance in these areas make solar power uneconomical (see Section 1c of the SM). For simplicity, we have based the PF estimation on fixed tracking PV systems on flat land. Solar yields can slightly differ (about 25% in both ways) for 1- or 2-axis PV tracking systems or for CSP systems^19^. See Table S5 in the SM for the assumed values of the parameters in Eq. (4) for the focus regions of this study.

4 $${{{\rho }_{e}}^{AEZ}={I}^{AEZ}\cdot {f}_{1}^{t} \cdot { f}_{2}\cdot {f}_{3}}^{AEZ}$$

5 $${{f}_{3}}^{AEZ}=GSR\cdot {PF}^{AEZ}$$

6 $${PF}^{AEZ}={(cos {\beta }^{AEZ}+\frac{sin {\beta }^{AEZ}}{\mathrm{tan}\left(66.55^\circ \cdot (\frac{\Pi }{180^\circ })-{\varnothing }^{AEZ}\right)})}^{-1}; \beta\,\mathrm{and }\,\varnothing\,\mathrm{in}\,\mathrm{radians}$$

Figure S6 of the SM defines the solar yield per AEZ. Note that this figure only represents the land inputs per unit of energy output. The capital inputs per unit of output depend only on *I*^*AEZ*^, *f*_*1*_^*t*^ and *f*_*2*_ and since capital costs tend to be larger than land costs, investors in solar energy tend to choose the location predominantly based on solar irradiance instead of the solar energy yield per land unit. Consistently exporting or importing large shares of solar energy between geographically and/or politically distinct regions faces both technical and geopolitical challenges. Therefore, we have chosen a conservative assumption that solar energy must be produced and consumed in the same geopolitical GCAM region.

### Impact of solar energy infrastructure on local carbon cycle

The impact of USSE infrastructures on local microclimates is a field in early research stages, although some case studies have been performed. In the case of solar energy on pastures in wet climates, a significant loss of carbon in vegetation and soils can be expected in the land below the infrastructure that is permanently blocked from sunlight, but the year-round carbon cycle in gap areas between rows of solar panels will be hardly affected^35^. However, in semi-arid pastures with wet winters, opposite effects are observed, and microclimates below panels seem to enhance vegetation growth^64^. Evidence from agrivoltaic systems show that year-round leaf growth below solar infrastructure is about 20% lower below solar modules, and that this effect is strongest in summer^65^. What can be concluded from these studies is that local microclimates depend on many factors, which are yet too uncertain to draw robust assumptions from. However, it also shows that the design and management of solar parks is of high importance for the carbon cycle in such parks. For example, higher placed modules are beneficial for vegetation growth below the modules, as it allows more sunlight to reach the vegetation. But a crucial aspect for local carbon cycles is the decision on how to manage the land below the solar energy infrastructure. That is why we have framed the uncertainty related to local carbon cycle impacts on management decisions, based on three different regimes identified through literature review:

- Land clearing: Clearing and grubbing of soil and roots, topsoil stripping and stockpiling, land grading and levelling, and soil compaction. Existing vegetation that supports habitat is removed and any other vegetation is often discouraged; weeds and other unwanted vegetation are generally managed with herbicides and by covering the ground with gravel; this is a common practice in various countries^41,66^. Modules are placed at ground level, which is cheaper, and the absence of vegetation avoids shading effects.
- Maintaining previous vegetation: Vegetation as in previous land uses is as much as possible maintained, so arable land stays arable and pastures stay pastures. All vegetation in previous land cover above 30 cm height, such as trees, bushes and high grass, will be removed such that the vegetation that is left is similar to that in pastures. Areas directly below solar modules are cleared for the construction phase, but weeds might grow after that phase. Modules are placed slightly higher to avoid potential shading from vegetation. This regime is based on a rationale of balancing cost minimisation (i.e. no seeding, no herbicides) with land conservation (i.e. minimising ecosystem disturbance).
- Pasture conversion: Irrespective of the previous land use, all land below and around the infrastructure is (re)seeded with grass before or right after the construction phase, and the land will be managed as pasture, allowing for extensive animal grazing around the solar modules^35^. Modules will be placed higher to allow small grazers to pass below^67^, and allowing some sunlight to reach vegetation below panels.

The impacts of each of these solarland management regimes on the local carbon cycle depend on the specific location, and the previous land use, and result from off-model calculations applied to the GCAM scenario outcomes which provide land cover changes per year, AEZ, and scenario. Section 2d of the SM gives full details on all applied assumptions and derived carbon cycle impacts.

### Use of non-competing space on rooftops and in deserts and dry scrublands

Rooftop space is often used for smaller scale PV systems and has the advantage of not competing for space with other uses and avoiding some of the losses related to electricity transmission and distribution. On the other side, rooftop spaces are often not optimal, and only about 2 to 3% of urbanized surface area can be used for PV systems with reasonable efficiencies (taking into account specific factors such as roof slopes and shadows between buildings)^1,21^. Taking these constraints into account, rooftop space is limited to 3% of expected urbanized land by 2050 (end year of the scenarios in this study) in each geo-political region, while non-optimality of rooftop space has been modelled through a supply curve which represents increasing capital costs for each additional space used for rooftop PV systems^68^.

Land that is not used and neither has potential for any other productive use from a human perspective, such as deserts and dry scrublands, can be suitable for solar energy. By default, deserts are exempted from land competition in GCAM, while only 10% of current scrublands are included in the land competition module in GCAM v4.3, taking into account both non-fertility of scrublands as well as the protected status of some of these land areas. The EU, Japan and South-Korea have limited amounts of deserts and scrublands (see Table S4 in the SM), and of which a significant share is protected^69^. Therefore, apart from the 10% of scrublands which enter by default into the land competition module, we assumed no additional availability of suitable deserts and scrublands for solar energy in these regions. For India, the pre-identified potential for PV and CSP capacity in identified “wasteland”^27^ is included to the model as an alternative to competitive land, under assumptions as specified in Section 1d of the SM.

Further background assumptions related to the modelling can be found in the SM.

## Supplementary Information

Supplementary Information.
