# Supplementary material for: The potential land requirements and related land use change emissions of solar energy
Source: Sci Rep. 2021 Feb 3;11:2907. doi: 10.1038/s41598-021-82042-5 (PMC7859221; doi:10.1038/s41598-021-82042-5)
Supplement: Supplementary file 1 — Supplementary Information. [file 41598_2021_82042_MOESM1_ESM.docx]

**The potential land requirements and related land use change emissions of solar energy**

Dirk-Jan Van de Ven^1^, Iñigo Capellan-Peréz^2^, Iñaki Arto^1^, Ignacio Cazcarro^1,3^, Carlos de Castro^2^, Pralit Patel^4^, Mikel Gonzalez-Eguino^1,5^

^1^ *Basque Centre for Climate Change (BC3), Edificio Sede 1-1, Parque Científico de UPV/EHU, Barrio Sarriena s/n, 48940 Leioa, Spain. Tel: +43 944014690*

^2^ *Research Group on Energy, Economy and System Dynamics, Escuela de Arquitectura, Av Salamanca, 18, University of Valladolid, 47014, Valladolid, Spain*

^3^ *ARAID-Aragonese Agency for Research and Development, Agrifood Institute of Aragon (IA2), Department of Economic Analysis, University of Zaragoza, Zaragoza, Spain*

^4^ *Joint Global Change Research Institute, Pacific Northwest National Laboratory, 5825 University Research Court, Suite 3500, College Park, MD 20740, United States*

*^5^ University of the Basque Country (UPV/EHU), Barrio Sarriena s/n, 48940 Leioa, Spain.*

Correspondence and requests for materials should be addressed to D.V. (email: dj.vandeven@bc3research.org)

| Contents: | Page |
| --- | --- |
|  |  |
| Section 1: Modelling assumptions   1. GCAM model 2. Solarland module specifications: Interaction with Energy module 3. Solarland module specifications: Interaction with Land use module 4. Update of historical solar electricity generation and costs in GCAM 5. Deserts and dry scrublands in India 6. Location-dependent grid costs for renewable energy 7. Management of RES variability in GCAM 8. Trade in bioenergy | **1**  **3**  **4**  **6**  **7**  **8**  **9**  **9** |
| Section 2: Scenario set-up   1. Regions 2. Penetration levels 3. Future efficiency of solar energy technologies 4. Land management regimes | **10**  **12**  **14**  **14** |
| Section 3: Scenario outcomes   1. Solar penetration scenarios 2. Land cover changes 3. CO_2_ payback period 4. Solarland as % of agricultural land by AEZ | **20**  **22**  **22**  **23** |
| References | **24** |

**Section 1: Modelling assumptions**

**a) GCAM.** For the study, we use the Global Change Assessment Model (GCAM) version 4.3^1^. GCAM is a dynamic-recursive model with technology-rich representations of the economy, energy sector, land use and water linked to a climate model that can be used to explore climate change mitigation policies including carbon taxes, carbon trading, regulations and accelerated deployment of energy technology. Regional population and labour productivity growth assumptions drive the energy and land-use systems employing numerous technology options to produce, transform, and provide energy services as well as to produce agriculture and forest products, and to determine land use and land cover. Using a run period extending from 1990 – 2100 at 5 year intervals, GCAM has been used to explore the potential role of emerging energy supply technologies and the greenhouse gas consequences of specific policy measures or energy technology adoption such as carbon capture and storage, bioenergy, hydrogen systems, nuclear energy, renewable energy technology, or energy use technologies in buildings, industry and transportation sectors. GCAM is a Representative Concentration Pathway (RCP)-class model. This means it can be used to simulate scenarios, policies, and emission targets from various sources including those from the Intergovernmental Panel on Climate Change (IPCC). Output includes projections of future energy supply and demand and the resulting greenhouse gas emissions, radiative forcing and climate effects of 16 greenhouse gases, aerosols and short-lived species, contingent on assumptions about future population, economy, technology, and climate mitigation policy.

Land use change (LUC) emissions in GCAM are calculated based on carbon release or uptake related to changes of the net land cover between each model simulation period. For each land cover, and each pre-defined Agro-Ecological Zone (AEZ; see Figure S1 for the distribution of land in AEZs for the focus regions of this study), assumptions on the amount of carbon sequestered in the vegetation and the soil are made (Table S1). The replacement of land cover with high carbon density in vegetation by land cover with lower density leads to direct LUC emissions, equal to the difference in carbon density. On the contrary, if the carbon sequestered in vegetation increases due to a net change in land cover, an Bertalanffy-Richards function^2^ is applied for the growth of sequestered carbon over an assumed maturity time, which is defined specifically for each land cover and AEZ. For example, this maturity time is set to 1 year for crops, but ranges between 30 and 100 year for forest, depending on the location. In the case of soil carbon, carbon sequestration or release due a land cover change occurs logarithmically over an assumed period. Generally, GCAM assumes that half of the change in soil carbon stocks will occur in 2 and a half years in tropical regions, and in 5 years in temperate regions, while the rate of change slows down when more time has passed since the land cover change took place.

Land cover changes in GCAM are driven by land use decisions made by land owners. Land owners choose between different land uses to maximise profit within their AEZ, depending on geographically varying yields, production costs and calibrated commodity prices. Such land use decisions are based on the logit model of sharing to avoid extreme “winner-takes-it-all” outcomes^3^. Note that commodities are assumed to be traded in a single, free trade global market thus each region sees a global price. A full description of the land module in GCAM is described in Wise and Calvin (2011)^4^, and a list of data sources and method are further described in Kyle et al. (2011)^5^.

Table S1: Assumed carbon stocks in soil and vegetation by type of land and AEZ, as assumed in GCAM land-use module^5^

|  | **Forest: Evergreen [1] [3][4]** | | **Forest:**  **Deciduous [3]** | | **Grassland: Steppe [2][3]** | | **Grassland: Savanna [2][4]** | | **Dense Shrubland [3]** | | **Open Scrubland [3]** | | **Arable/Crops [3]** | |
| --- | --- | --- | --- | --- | --- | --- | --- | --- | --- | --- | --- | --- | --- | --- |
| **AEZ** | **Soil [1]** | **Veg.** | **Soil** | **Veg.** | **Soil** | **Veg.** | **Soil** | **Veg.** | **Soil** | **Veg.** | **Soil** | **Veg.** | **Soil** | **Veg. [5]** |
|  | *Assumed carbon stocks in kg C/m^2^ (annual average)* | | | | | | | | | | | | | |
| ***01*** | 9.8 | 20 | 9.8 | 14 | 6 | 0.4 | 9.5 | 2.5 | 6.9 | 5.5 | 6.9 | 2.7 | 3 | 0.05-1.5 |
| ***02*** | 9.8 | 20 | 9.8 | 14 | 7.5 | 0.7 | 9.5 | 2.5 | 6.9 | 5.5 | 6.9 | 2.7 | 4 | 0.05-1.5 |
| ***03*** | 9.8 | 20 | 9.8 | 14 | 9 | 0.7 | 9.5 | 2.5 | 6.9 | 5.5 | 6.9 | 2.7 | 5 | 0.05-1.5 |
| ***04*** | 9.8 | 20 | 9.8 | 14 | 10.5 | 0.7 | 9.5 | 2.5 | 6.9 | 5.5 | 6.9 | 2.7 | 6 | 0.05-1.5 |
| ***05*** | 9.8 | 20 | 9.8 | 14 | 12 | 0.85 | 9.5 | 2.5 | 6.9 | 5.5 | 6.9 | 2.7 | 6 | 0.05-1.5 |
| ***06*** | 9.8 | 20 | 9.8 | 14 | 13.5 | 1 | 9.5 | 2.5 | 6.9 | 5.5 | 6.9 | 2.7 | 6 | 0.05-1.5 |
| ***07*** | 7.1-13.4 | 15.7 | 13.4 | 13.5 | 8.5 | 0.6 | 9.5 | 2.5 | 6.9 | 5.5 | 6.9 | 2.7 | 6 | 0.05-1.5 |
| ***08*** | 7.1-13.4 | 15.7 | 13.4 | 13.5 | 10 | 0.7 | 9.5 | 2.5 | 6.9 | 5.5 | 6.9 | 2.7 | 7 | 0.05-1.5 |
| ***09*** | 7.1-13.4 | 15.7 | 13.4 | 13.5 | 11.5 | 0.7 | 9.5 | 2.5 | 6.9 | 5.5 | 6.9 | 2.7 | 8 | 0.05-1.5 |
| ***10*** | 7.1-13.4 | 15.7 | 13.4 | 13.5 | 13 | 0.7 | 9.5 | 2.5 | 6.9 | 5.5 | 6.9 | 2.7 | 9 | 0.05-1.5 |
| ***11*** | 7.1-13.4 | 15.7 | 13.4 | 13.5 | 14.5 | 0.85 | 9.5 | 2.5 | 6.9 | 5.5 | 6.9 | 2.7 | 9 | 0.05-1.5 |
| ***12*** | 7.1-13.4 | 15.7 | 13.4 | 13.5 | 16 | 1 | 9.5 | 2.5 | 6.9 | 5.5 | 6.9 | 2.7 | 9 | 0.05-1.5 |
| ***13*** | 20.6 | 9 | 20.6 | 9 | 11 | 0.6 | 9.5 | 2.5 | 6.9 | 5.5 | 6.9 | 2.7 | 9 | 0.05-1.5 |
| ***14*** | 20.6 | 9 | 20.6 | 9 | 12.5 | 0.7 | 9.5 | 2.5 | 6.9 | 5.5 | 6.9 | 2.7 | 10 | 0.05-1.5 |
| **15** | 20.6 | 9 | 20.6 | 9 | 14 | 0.7 | 9.5 | 2.5 | 6.9 | 5.5 | 6.9 | 2.7 | 11 | 0.05-1.5 |
| **16** | 20.6 | 9 | 20.6 | 9 | 15.5 | 0.7 | 9.5 | 2.5 | 6.9 | 5.5 | 6.9 | 2.7 | 12 | 0.05-1.5 |
| **17** | 20.6 | 9 | 20.6 | 9 | 17 | 0.85 | 9.5 | 2.5 | 6.9 | 5.5 | 6.9 | 2.7 | 12 | 0.05-1.5 |
| **18** | 20.6 | 9 | 20.6 | 9 | 18.5 | 1 | 9.5 | 2.5 | 6.9 | 5.5 | 6.9 | 2.7 | 12 | 0.05-1.5 |
| *Soil: Carbon stocks in and below soil.*  *Veg.: Carbon stocks in above-ground vegetation*  [1] Carbon stocks in Evergreen forest soil in AEZ 07 to 12 depends on relative amount of Broadleaf (= 71 kg C/m^2^) & Needleleaf (= 134 kg C/m^2^) forest  [2] Carbon stocks in “Pasture” soil (Figure S4) is assumed equal to carbon stocks in grassland for each region & AEZ. Carbon stocks by vegetation in pastures is assumed to be equal as in steppe grassland in each AEZ.  [3] Interpreted from Houghton (1999)^6^ by Kyle et al (2011)^5^  [4] Interpreted from King et al (1997)^7^ by Kyle et al (2011)^5^  [5] Depending on the amount of carbon stocks in each type of crop, specified by region and AEZ. Be default, value is 0.3 for unused arable land. | | | | | | | | | | | | | | |


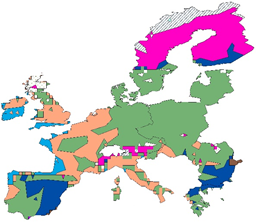

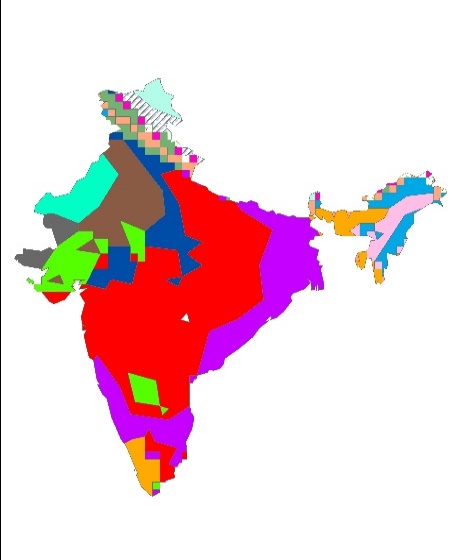

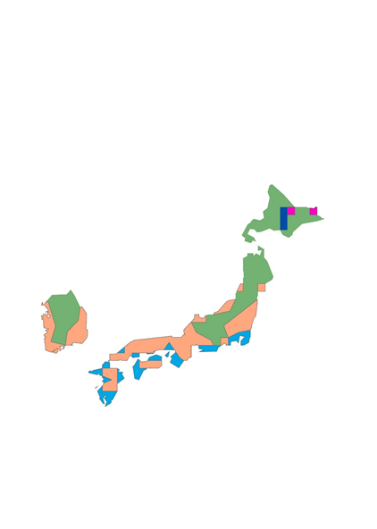

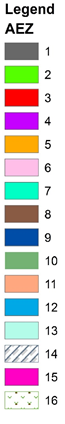


Figure S1: Overview of Agro-Ecological Zones (AEZs) in the focus regions of this study (see section 2a for further elaboration on regional choice). Note that there is a regional breakdown between “EU-15” (representing the EU up to 2004) and “EU-12” (representing countries that entered the EU from 2004 onwards, except Croatia) and between Japan and South-Korea. This means that if the same AEZ overlaps over these separated regions (e.g. AEZ 10 in EU-27), they are treated as separated land regions. Source: Authors´ own elaboration with the Arc GIS 10.5.1 Desktop (Esri) software.

**b) Solarland module specifications: Interaction with Energy module.** For the purpose of this study, a “solarland” module has been developed for the Global Change Assessment Model (GCAM). This module specifies the requirement of land for land-based utility scale solar energy (USSE). This USSE, either in the form of PV or CSP, only enters into the energy system through electric power generation. At the electricity consumption point, centrally generated electricity competes with distributed generation, dominated by rooftop solar systems. Figure S2 shows how the energy system in GCAM demands solarland through this module.

Solar energy produced on solarland in different Agro Ecological Zones (AEZs; see Figure S1) compete for their market share within the solar subsector of electricity, separately within each GCAM region. Competition between different technologies in most energy sectors in GCAM is modelled through the Modified Logit model^8^ (see equation 1 with *s* and *p* representing respectively the relative share and price of each technology *i*, and *a* and *y* representing respectively the “shareweight” of each technology and the “logit exponent” of the whole sector). However, the scenarios for this study that stimulate solar technologies (including rooftop solar) to represent up to 80% of the electricity mix imply large subsidies, bringing costs down to around zero in some cases. Therefore, competition between different AEZs in the solar market, and also between utility scale power (“Electric Power Generation” in Figure S2) or distributed rooftop solar, goes through the Logit model (see equation 2), which behaves better for values close to zero^1,9^.

$s_{i}= \frac{a_{i}p_{i}^{\gamma}}{\sum_{j=1}^{N} a_{i}p_{i}^{\gamma}}$ (1)

$s_{i}= \frac{a_{i}exp(\beta p_{i})}{\sum_{j=1}^{N} a_{i}exp(\beta p_{i})}$ (2)

The shareweight of each AEZ, defining a pre-determined technology preference, has been defined by the relative share of the total land area of that AEZ within every region. For example, this means that if the Levelized cost of electricity (LCOE) in each AEZ of a certain region were exactly equal, solar energy production would be evenly spread over the region with each AEZ hosting the share which corresponds to its share of total land area. Similarly, the shareweight of solar energy from “wasteland” in India (see section SM2d) equals the total relative size of such areas in India^10^, and the shareweight of rooftop solar is equal to the relative share of urban land by 2010 and increases proportionally with simulated population increase until 2050.


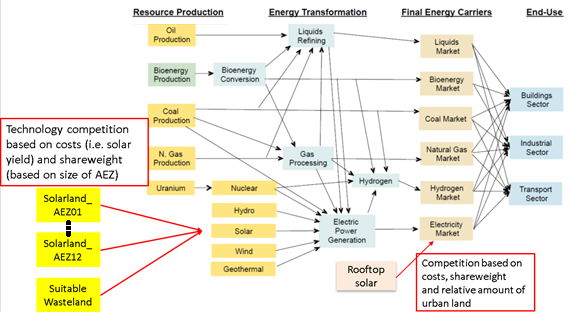


Figure S2: Representation of how the solarland module is included within the energy system in GCAM. Adapted from: ^1^

**c) Solarland module specifications: Interaction Land use module.** The solarland module introduces competition of solarland with other land uses. Figure S3 shows how the land competition between different land-uses is structured in GCAM, and how the solarland module has been inserted in the “Crops” node. Rooftop solar is assumed to enter in urban land, which is exogenous in GCAM, while deserts and dry scrubland that are suitable for solar energy, but not for crops, are also considered exogenous. Those land-use categories (e.g. corn, wheat, bioenergy, solarland) belonging to the same node (crops in this example) are assumed to compete more directly with else other than with those land-uses in other nodes (e.g. forest or pasture).


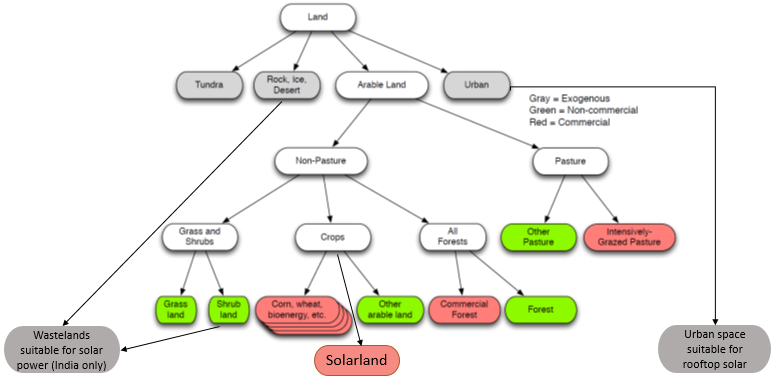


Figure S3: Representation of how the solarland module is included in the land competition structure of GCAM. Land uses in red compete for land, land uses in grey (exogenous) and green (endogenous) do not. Adapted from JGCRI (2017) ^1^

A comparison of known geolocations of solar power plants with land cover data obtained from satellite views indicates that, in the three focus regions of this study (elaborated in section SM2a), solar energy is indeed often installed in land cover types that fall under arable land in the GCAM land module: cropland and bare areas (see Table S2). While this data only covers a small subset of total solar power plants, this finding is supported by the literature in the case of various European countries^11–14^, and makes sense from an economic point of view as cropland is generally found to be most productive type of land for hosting solar energy^15^. However, Table S2 also indicates that an important share of solar installations are built on grassland and scrubland, and literature confirms this finding for areas where overall land cover is dominated by such land cover types^16,17^. The land node construction shown in Figure S3 takes such effects into account, as beyond the modelled “preference” for arable land, allocation decisions for USSE depend on the relative profitability of other land uses. In AEZs where some non-arable land cover (e.g. pasture, scrubland, forest) is abundant, the (potential) marginal profitability of that land is usually relatively low, increasing the probability that USSE is allocated in this abundant land category.

Table S2: Allocation of current solar power capacity in the Global Database of Power Plants by land cover type

|  | **Total Capacity (MW)** | | |  | **% of identified capacity** | | |
| --- | --- | --- | --- | --- | --- | --- | --- |
|  | *EU** | *India* | *Japan & S Korea* |  | *EU** | *India* | *Japan & S Korea* |
| *Postflooding or irrigated croplands (or aquatic)* | 322.90 | 968.40 | - |  | 1.5% | 38.3% | 0.0% |
| *Rainfed croplands* | 6,846.44 | 173.80 | 922.90 |  | 31.5% | 6.9% | 44.3% |
| *Mosaic cropland (50-70%) / vegetation (grassland/shrubland/forest) (20-50%)* | 4,321.37 | 118.50 | 307.20 |  | 19.9% | 4.7% | 14.7% |
| *Mosaic vegetation (grassland/shrubland/forest) (50-70%) / cropland (20-50%)* | 1,942.05 | 204.00 | 39.10 |  | 8.9% | 8.1% | 1.9% |
| *Closed to open (>15%) broadleaved evergreen or semideciduous forest (>5m)* | 106.97 | 5.00 | - |  | 0.5% | 0.2% | 0.0% |
| *Closed (>40%) broadleaved deciduous forest (>5m)* | 898.18 | - | 47.80 |  | 4.1% | 0.0% | 2.3% |
| *Open (15-40%) broadleaved deciduous forest/woodland (>5m)* | - | - | - |  | 0.0% | 0.0% | 0.0% |
| *Closed (>40%) needleleaved evergreen forest (>5m)* | 189.37 | - | 74.00 |  | 0.9% | 0.0% | 3.6% |
| *Open (15-40%) needleleaved deciduous or evergreen forest (>5m)* | 195.56 | - | 2.00 |  | 0.9% | 0.0% | 0.1% |
| *Closed to open (>15%) mixed broadleaved and needleleaved forest (>5m)* | 352.75 | - | 162.90 |  | 1.6% | 0.0% | 7.8% |
| *Mosaic forest or shrubland (50-70%) / grassland (20-50%)* | 1,052.00 | - | - |  | 4.8% | 0.0% | 0.0% |
| *Mosaic grassland (50-70%) / forest or shrubland (20-50%)* | 1,001.77 | 345.00 | 1.50 |  | 4.6% | 13.6% | 0.1% |
| *Closed to open (>15%) (broadleaved or needleleaved, evergreen or deciduous) shrubland (<5m)* | 19.58 | - | - |  | 0.1% | 0.0% | 0.0% |
| *Closed to open (>15%) herbaceous vegetation (grassland, savannas or lichens/mosses)* | 2,380.04 | 302.00 | 72.80 |  | 11.0% | 11.9% | 3.5% |
| *Sparse (<15%) vegetation* | 842.47 | 30.00 | 21.10 |  | 3.9% | 1.2% | 1.0% |
| *Closed to open (>15%) broadleaved forest regularly flooded (semipermanently or temporarily)* | - | - | - |  | 0.0% | 0.0% | 0.0% |
| *Closed (>40%) broadleaved forest or shrubland permanently flooded* | - | - | - |  | 0.0% | 0.0% | 0.0% |
| *Closed to open (>15%) grassland or woody vegetation on regularly flooded or waterlogged soil* | 96.50 | - | - |  | 0.4% | 0.0% | 0.0% |
| *Artificial surfaces and associated areas (Urban areas >50%)* | 710.57 | 32.00 | - |  | 3.3% | 1.3% | 0.0% |
| *Bare areas* | 304.40 | 330.80 | - |  | 1.4% | 13.1% | 0.0% |
| *Water bodies* | 121.98 | 20.00 | 431.80 |  | 0.6% | 0.8% | 20.7% |
| *Permanent snow and ice* | - | - | - |  | 0.0% | 0.0% | 0.0% |
| *No data (burnt areas, clouds)* | - | - | - |  | 0.0% | 0.0% | 0.0% |
| *Total identified* | 21,704.90 | 2,529.50 | 2,083.10 |  |  |  |  |
| *Total installed (2017*^18^*)* | 107150 | 18300 | 54600 |  |  |  |  |
| *% coverage* | 20% | 14% | 4% |  |  |  |  |
| *Geolocation of solar capacity was obtained through the Global Database of Power Plants: https://www.wri.org/publication/global-power-plant-database. The geolocation data has been contrasted with land cover data from the Globcover Portal to match each solar power project with the land cover category as defined in this data layer: http://due.esrin.esa.int/page_globcover.php. As the last row shows, these data only refer to a relatively small subset of total solar energy capacity, and predominantly include large solar power plants. Therefore, these data should not be interpreted as the absolute land cover distribution of solar power plants, but just as an indicative distribution.*  ** There was no data for all EU countries, so the values in this column represent identified capacities in Czech Republic, Denmark, France, Germany, Italy, Poland, Portugal, Spain and the United Kingdom.* | | | | | | | |

Indirect LUC emissions related to the penetration of solar energy, e.g. by displacing cropland to other areas where it leads to deforestation or by occupying land that would otherwise have been afforested, are calculated through the default assumptions on carbon stocks in GCAM (see Table S1). However, direct LUC emissions from the conversion of other land to solarland depend on various factors, such as the previous land cover and the way solarland is managed from the point of construction onwards. For the purpose of this study, we have therefore formulated three solarland management scenarios, driving the impact of USSE on local carbon cycles. All assumptions made in these scenarios are provided in section SM2d.

Like in other studies^19–21^, some limitations are implemented for the possibility of installing utility scale solar capacity in some AEZs, primarily based on solar resources. Utility Scale PV capacity is excluded for AEZ 13 to 16 (see Figure S1), which are located in far northern areas with very little radiation and extreme seasonal variability or in highly mountainous areas. In mountainous areas, solar irradiance can be relatively high, but high slopes prevent the installations of utility scale PV systems. Instead, rooftop PV systems in these areas are implicitly included in our estimate for total rooftop potential in each region. CSP technologies only use direct normal component of sunlight intensity, which is limited or subject to high variability in many regions, making CSP systems uneconomical in such regions^19,20,22^. Therefore, we limited the potential of CSP to north western India (AEZ 2, 7, 8 and 9) and some parts of southern Europe (AEZ 4, 8 and 9 and a small part of AEZ 10 in Western Europe).

**d) Update of historical solar electricity generation and costs in GCAM.**

Version 4.3 of the GCAM model is calibrated until 2010. This means that by structure, the technological “preferences” of 2010 are remembered into the future. This has significant implications for solar energy. Compared to no-policy scenarios in GCAM, the actual output of solar electricity in 2015^23^ is about 4 times higher in the EU and 10 times higher in Japan. This increased “preference” has important implications for actual and future land use for solar power in all scenarios. Therefore, to take these developments into account, we have calibrated the electricity mix and total electricity consumption for the EU, India, Japan and South-Korea for 2015 following the IEA energy balance database^23^. Preferences for all solar technologies are modelled to converge by 2050. Nuclear power in Japan, which is below 1% in 2015 due to the Fukushima incident in 2011, is modelled to return step by step such that it represents 20% of Japanese electricity by 2030, as is projected in the Japanese NDC submission^24^.

See Table S3 for the electricity mix we calibrated for 2015, including an estimated distribution between land-based and rooftop-based solar energy production. More detailed sources are used to estimate the share of land-based and rooftop-based PV in the EU and Japan^25,26^. For India and South-Korea, where the penetration of solar PV in 2015 was insignificant, equal shares of utility-scale and residential PV are assumed in 2010, and the shares of 2015 follow from model simulation. The simulated domination of land-based solar in both countries by 2015 is confirmed by real-world observations^27^. On a global scale, rooftop based PV accounted for around 35% of solar PV capacity in 2015^28^ (the rooftop share of solar power output is probably lower than that, given the lower overall efficiency of rooftop versus land-based systems^29^). However, country-specific distributions between rooftop and land-based PV are harder to find, although a distribution of installed capacity based on system size is more accessible. Based on data on system size in the EU and Japan, we considered the cut-off size between land-based and rooftop-based PV to be 500 kWp. In reality, there will be land-based systems of less than 500 kWp, and industrial/commercial rooftop systems of more than 500 kWp, but it is expected to be a good overall estimate.

Capital costs of solar energy systems are expected to decrease in the future, while the efficiency of the technology is expected to increase (see section SM2c). For the future costs of solar energy projects, GCAM considers on learning-curve models^30^. The higher estimates for overnight capital costs of residential PV and utility-scale PV as reported by the IEA^31^ have been used for 2015, while median estimates have been used for the costs of CSP. For future periods, we applied the original GCAM learning curve until 2050^30^. See Figure S4 for the assumed cost evolution of the solar technologies used. However, these assumptions do not affect the results given that the level of total solar penetration is imposed and both PV and CSP technologies are assumed to have the same power density.

| 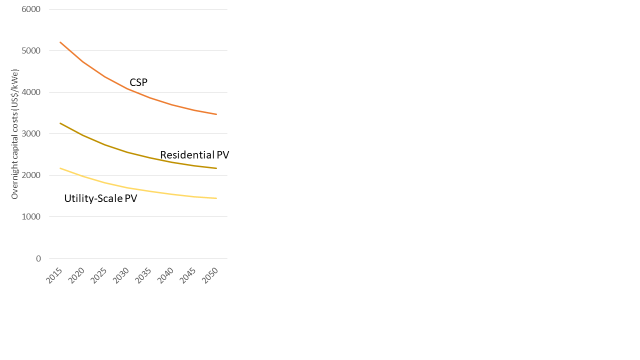  Figure S4: assumed overnight capital costs of solar technologies | Table S3: Calibrated 2015 electricity mix of regions/countries in this study   \| % of total electricity in 2015 \| \| \| *EU-27* \| *India* \| *Japan* \| *South Korea* \| \| --- \| --- \| --- \| --- \| --- \| --- \| --- \| \| *Fossil energy* \| \| \| 42.60% \| 81.89% \| 82.15% \| 67.28% \| \| *Bioenergy* \| \| \| 6.24% \| 1.92% \| 3.98% \| 0.57% \| \| *Nuclear* \| \| \| 26.58% \| 2.71% \| 0.93% \| 29.80% \| \| *Other renewables* \| \| \| 21.23% \| 13.08% \| 9.51% \| 1.65% \| \| ***Land-based PV*** \| \| \| **1.95%** \| **0.37%** \| **1.95%** \| **0.65%** \| \| ***Rooftop-based PV*** \| \| \| **1.23%** \| **0.04%** \| **1.49%** \| **0.05%** \| \| ***CSP*** \| \| \| **0.17%** \| **0.00%** \| **0.00%** \| **0.00%** \| \|  \|  \|   *Source:* ^23,25,26^ |
| --- | --- | --- | --- | --- | --- | --- | --- | --- | --- | --- | --- | --- | --- | --- | --- | --- | --- | --- | --- | --- | --- | --- | --- | --- | --- | --- | --- | --- | --- | --- | --- | --- | --- | --- | --- | --- | --- | --- | --- | --- | --- | --- | --- | --- | --- | --- | --- | --- | --- | --- | --- | --- | --- | --- | --- | --- | --- | --- | --- |

**e) Deserts and dry scrublands in India.** In contrast to the EU, Japan and South-Korea and despite its high population density, India has plenty of land identified by its central government as “wasteland”: land that is not used for human purposes, such as desert- and scrubland, degraded pasture and degraded cropland, old mining grounds and various other land categories^10^. Installing solar power on such unproductive land avoids competition with agricultural land. By excluding land with an average slope greater than 2.1% and land with low solar irradiance, Mahtta et al.^19^ estimate the maximum amount of solar power capacity that could be installed on such wasteland to be 6000 GW for PV and 2500 GW for CSP. Using a 20% average capacity factor, this translates into 37.84 EJ per year and 15.77 EJ per year of electricity of PV and CSP respectively.

Although this land is currently considered wasteland by the central government, some of this land could potentially be turned into cropland or grazing land by, for example, chemical fertilisation. GCAM assumes that 10% of current grass- and scrubland could potentially overcome physical and bureaucratic limitations to be turned into commercial land (cropland, grazing land or commercial forest). Since the purpose of this study is to measure the impacts on land competition, we estimate the overlap between wasteland that could become commercial and the part that is suitable for solar power^19^. We estimate that 13.4% of the solar power potential on wasteland will be on land that could also be turned into other commercial land uses according to current GCAM assumptions. Since this 13.4% will already be included in the land competition module of GCAM, we modelled the remaining 86.6% of the solar power potential as an alternative “resource” that can host solar power capacity without entering into land use competitions.

However, due to the limitations of solar capacity in desert- and scrubland, such as grid proximity, water availability and remoteness from inhabited places, we assume that the construction and wiring costs in the most remote parts of these areas will be significantly higher. Since the most remote wastelands in India, located in Rajasthan, are around 350 km away from the nearest grid station, we calculate that additional investment in HVDC required for solar power plants in such remote areas can increase the total costs of such projects by up to 16% (own calculations based on data from Breyer et al., 2015^32^). Additionally, we can expect significant additional construction costs for such projects, as temporary villages would have to be developed for construction workers. Data on such costs are hard to find, but we assume that total construction costs (18% of total costs for non-remote large scale solar power projects in 2014^26^) could increase up to 50%, adding another 9% to the total costs of solar power projects in the most remote areas in India. On top of that, for CSP projects, the lack of cooling water could require air cooling, which consumes 7-9% of the produced electricity, while hybrid air/water cooling in the case of some water availability requires 5% of the produced electricity^33^. Therefore, we assume that the cost of CSP in deserts and dry scrublands could increase by up to 9% of the installation costs due to water scarcity (see eq. 11 in ^29^), and the maximum potential CSP output in such land drops by an average of 5% due to this limit. See Figure S5 for the total potential solar electricity on land that is classified as wasteland by the Indian government and the extra costs per unit of output due to physical limits. Since these additional costs are highly uncertain, we have performed tests to steepen and flatten the cost curve (assuming a maximum construction and wiring cost increase of respectively 200% and 50% instead of 100%) for solar energy in deserts and dry scrublands, and observed only marginal changes in the relative amount of solar energy installed on such land (< 5% difference).

| \| PV \| CSP \|  \| \| --- \| --- \| --- \| \| 6,000 \| 2,500 \| GW^19^ \| \| 37.84 \| 15.77 \| max EJ (20% average capacity factor) \| \| 32.76 \| 13.65 \| max EJ after “wasteland” correction (86.6%) \| \| 32.76 \| 12.97 \| max EJ after distracting air cooling inefficiency CSP \| |  |
| --- | --- | --- | --- | --- | --- | --- | --- | --- | --- | --- | --- | --- | --- | --- | --- | --- |
| Figure S5: Potential output and additional costs in $(1975) (standard cost unit in GCAM) per EJ for PV and CSP installed in Indian Wasteland. Average costs for solar electricity in India in 2015 are estimated to be 12 $(1975) per GJ in this study. | |

**f) Location-dependent grid costs for renewable energy.** The best conditions for renewable energy such as wind and solar are not necessarily close to demand hubs. For scenarios modelled in this study, strong convergence of solar capacity to areas with high solar resources within a certain region (i.e. Southern Europe, North West India, see Figure S6), but which are not necessarily close to demand hubs, should increase total grid costs of the energy system, as such grid requirements can significantly change the optimal spread over solar energy over a certain area^34^. For example, in a study where 80% of European electricity consumption comes from renewable energy of which one fourth would be imported from Northern Africa, about 10% of new investments would be dedicated to grid expansion^35^.

To take into account such grid costs related to a geographically unbalanced output of solar energy within a region, equation 3 represents the assumed additional grid costs per AEZ (C^AEZ^) as a function of the relative solar energy output in this AEZ. Since grid requirements also depend on the geographical distribution of energy demand, equation 4 shows how the “geo multiplier” per AEZ depends on the relative amount of urban land in that AEZ, which serves as a proxy for electricity demand within each AEZ. Due to this formula, grid costs would be limited if solar capacity is installed in AEZs with high electricity demand. On the other hand, grid costs increase exponentially if all solar capacity is being installed in the same AEZ, and costs would be even higher if only a small part of electricity demand would be located in this AEZ. Costs are calibrated such that total grid costs at high penetration scenarios in the EU represent about 5% of the total LCOE with a balanced geographical distribution of solar energy generation and up to 15% with an unbalanced geographical distribution of solar energy (i.e., all solar energy installed in the AEZ 8 and 9; Figure S1). These assumptions are expanded to India, Japan and South-Korea.

$C_{t}^{AEZ}=\frac{{output}_{t}^{AEZ}*{geo\_multiplier}^{AEZ}}{{Total electricity}_{t}-{output}_{t}^{AEZ}}$ (3)

${geo\_multiplier}^{AEZ}= \left( \frac{4*Total Urbanland}{{Urbanland}^{AEZ}} \right)^{0.3}$ (4)

**g) Management of RES variability in GCAM.** At high penetration rates of intermittent energy sources such as wind and solar in the electricity system, storage or back-up systems are required to meet electricity demand at all hours. Therefore, GCAM assumes increasing back-up requirements with intermittent energy penetration. The cost of a backup technology is modelled on a gas turbine with a 5% capacity factor. To avoid back-up costs, solar and wind energy can be combined with on-site capital-intensive storage systems (i.e., electric batteries)^36^.

Both back-up and storage systems increase the total land requirements for solar energy. In the case of back-up systems, total electricity production increases for the same level of final electricity demand, which means that more solar energy has to be installed to reach a certain penetration level, and consequently more land is required to host the additional solar energy. For example, with a target of 80% solar in the electricity mix with 10 EJ electricity demand, 8 EJ solar would be produced. However, given that gas backup increases total required electricity production by 0.4 EJ (5%), therefore, a total of 8.32 EJ of solar would be required to actually reach 80% of total electricity production, ultimately increasing the land requirements for solar energy. The penetration levels explained in section SM2b include back-up electricity. Storage systems suffer full-cycle efficiency losses. Therefore, more solar capacity is required to make up for these losses, hence increasing land use. To take these losses into account, we have assumed a 15% lower land use efficiency for solar energy with storage (based on a 75% average round-trip efficiency of storage systems and 60% of solar electricity being stored)^37^.

**h) Trade in bio-energy.** For bio-energy, we separated domestic and imported resources. In GCAM v4.3, there is a global market for bio-energy. Although it makes economic sense in various occasions to trade bio-energy resources over large distances due to large differences in production costs, the transport costs for imported biomass are on average higher than for regionally produced bio-energy^38^. In order to control the origin of biomass production and to represent the bio-energy market more realistically, we have separated domestic bio-energy production in the three focus markets (EU-27, India, Japan + South-Korea). Following Hamelinck et al^38^, we assume that transport costs of imported bio-energy, valued at 3.15 $(2015) per GJ are 33% higher than transport costs for domestically produced bio-energy ($2.36 per GJ), while keeping average transport costs of total bio-energy consumption in 2010 equal to other regions and as assumed by default ($2.63 per GJ). This change is predominantly important for those scenarios where bio-energy technologies are modelled as the dominant renewable energy technology (See methods section).

**Section 2: Scenario set-up**

**a) Regions.** For this study, we limited our study to the European Union, India and jointly Japan and South-Korea, because of two main reasons:

- Results on land use impacts were expected to be more relevant^29^
- A negligible or, in the case of India, well quantified potential of solar energy in so-called “wasteland"^19^ (deserts and dry scrublands unsuitable for other human purposes), reduces uncertainty about the outcomes of this study.

Notwithstanding, these regions differ in terms of solar irradiation, latitude, land cover, energy use per capita and energy system. Table S4 shows some specific conditions of these three regions compared to United States and China, where land availability frictions will be likely of less intensity. Desert and scrubland areas are very limited in the European Union (EU), while the region aims for ambitious renewable energy targets, already in the medium term. India has a fast population growth and an even faster growing use of energy per unit of land, while urban space per capita is very limited and the majority of its land is used for sown or potential cropland. Japan and South Korea are characterized by very high energy use within a limited land surface, which consists mostly out of forests. In contrast, countries such as the United States and China have either a lower population density, energy use density, higher urban space per capita or consist for a significant share out of deserts and scrubland that could potentially host solar power without involving land competition. Recent observations indeed show that about half of solar energy in California is installed in either urban areas or scrubland, avoiding competition with other land uses that are productive from a human point of view (despite the potential high biodiversity value of the latter)^16^. Therefore, we concluded that extending the study to countries like the United States and China would involve too much uncertainty with respect to the impact of solar energy on land competition.

Table S4: Regional characteristics relevant for land requirements of solar energy. In bold the characteristics that make each of the chosen regions relevant for this study.

| Focus  Region: | Population density (inhabitants per km2) | | (Final) Energy use density  (TJ per km2) | | Urban space per capita (m^2^) | Share of land occupied by^39^: | | | Solar in electricity mix^40^ | Renewable electricity target  (approx.)** |
| --- | --- | --- | --- | --- | --- | --- | --- | --- | --- | --- |
|  |  |  |  |  |  | Desert/ scrub-land | Arable land | Forest |  |  |
|  | 2010 | 2050* | 2010 | 2050* | 2010 | 2010 | | | 2015 | 2030 |
| European Union | 128 | 119 | 16.9 | 17.4 | 209 | **4.0 %** | 28.6 % | 44.9 % | 3.3 % | **47.5 %**^24,41^ |
| India | 405 | **521** | 9.6 | **31.7** | **18** | 9.7 % | **55 %** | 24.2 % | 0.4 % | 24 %^24^* |
| Japan and S-Korea | 429 | 399 | **70** | **56** | **77** | **2.1 %** | 12 % | **81.5 %** | 2.5 % | 22 %^24,42^ |
| *USA* | *36* | *49* | *9.7* | *10.2* | *595* | *8.6 %* | *18.1 %* | *36.5 %* | *0.8 %* |  |
| *China* | *144* | *136* | *10.8* | *12.8* | *41* | *15.7%* | *13.5 %* | *18.6 %* | *0.8 %* |  |
| * Own estimates for 2050 are taken from a reference scenario run by GCAM based on SSP2 parameters^43^. India target of 40% of electricity capacity based on non-fossil resources translate to 30% of non-fossil electricity output of which one fifth (6% of total electricity) is nuclear energy, based on a GCAM reference scenario with SSP2 parameters.  ** Based on initial renewable energy targets as proposed in Paris Agreement INDCs, either explicit or implicit. | | | | | | | | | | |

Higher solar irradiance translates to more energy output from the same panel and thus less land requirements per unit of energy. On the other hand, higher latitude translates to more shading of either PV panels or CSP mirrors, increasing the required land area for USSE to prevent self-shading of panels or mirrors^44^. Both solar irradiance and latitude strongly vary between the northern and Mediterranean part of the EU, while conditions in Japan and South-Korea are more homogenous and are comparable to the Mediterranean part of Europe. With a higher Direct Normal Irradiance (DNI) and lower latitude, conditions in India are considerably more favourable, with the north western part of the country being recognized as a solar energy hotspot^19^. Table S5 shows the estimated electricity output for the focus regions in this study, per AEZ (see Figure S1), and depending on irradiance and other inputs. Figure S6 displays the geographical differences in terms of solar irradiation, and the calculated average solar electricity yields by AEZ. See the Methods section (main article) how DNI and latitude influence land requirements of USSE in the three regions.

Table S5: assumptions on irradiance, efficiency, performance, land-occupation ratio and electricity output, by region and AEZ.

| **Geopolitical region** | **AEZ** | **Solar irradiance *(I)* (kWh/m2/day)** | ***f_1_*** | | ***f_2_*** | ***f_3_*** | **Electricity output (kWh/m2/day)** | |
| --- | --- | --- | --- | --- | --- | --- | --- | --- |
|  |  |  | **2010** | **2050** |  |  | **2010** | **2050** |
| EU-12 | 8 | 3.59 | 0.12 | 0.2 - 0.28 | 0.65 | 0.28 | 0.08 | 0.13 - 0.18 |
| EU-12 | 9 | 3.69 | 0.12 | 0.2 - 0.28 | 0.65 | 0.30 | 0.09 | 0.14 - 0.2 |
| EU-12 | 10 | 2.99 | 0.12 | 0.2 - 0.28 | 0.65 | 0.20 | 0.05 | 0.08 - 0.11 |
| EU-12 | 11 | 3.32 | 0.12 | 0.2 - 0.28 | 0.65 | 0.26 | 0.07 | 0.11 - 0.16 |
| EU-15 | 4 | 4.79 | 0.12 | 0.2 - 0.28 | 0.65 | 0.38 | 0.14 | 0.24 - 0.33 |
| EU-15 | 8 | 4.59 | 0.12 | 0.2 - 0.28 | 0.65 | 0.37 | 0.13 | 0.22 - 0.31 |
| EU-15 | 9 | 3.83 | 0.12 | 0.2 - 0.28 | 0.65 | 0.27 | 0.08 | 0.14 - 0.19 |
| EU-15 | 10 | 3.35 | 0.12 | 0.2 - 0.28 | 0.65 | 0.24 | 0.06 | 0.1 - 0.14 |
| EU-15 | 11 | 3.29 | 0.12 | 0.2 - 0.28 | 0.65 | 0.25 | 0.06 | 0.11 - 0.15 |
| EU-15 | 12 | 3.11 | 0.12 | 0.2 - 0.28 | 0.65 | 0.24 | 0.06 | 0.1 - 0.14 |
| India | 1 | 5.18 | 0.12 | 0.2 - 0.28 | 0.65 | 0.51 | 0.21 | 0.34 - 0.48 |
| India | 2 | 5.21 | 0.12 | 0.2 - 0.28 | 0.65 | 0.54 | 0.22 | 0.36 - 0.51 |
| India | 3 | 5.14 | 0.12 | 0.2 - 0.28 | 0.65 | 0.55 | 0.22 | 0.37 - 0.51 |
| India | 4 | 5.01 | 0.12 | 0.2 - 0.28 | 0.65 | 0.56 | 0.22 | 0.36 - 0.51 |
| India | 5 | 4.78 | 0.12 | 0.2 - 0.28 | 0.65 | 0.56 | 0.21 | 0.35 - 0.49 |
| India | 6 | 4.33 | 0.12 | 0.2 - 0.28 | 0.65 | 0.50 | 0.17 | 0.28 - 0.39 |
| India | 7 | 5.03 | 0.12 | 0.2 - 0.28 | 0.65 | 0.48 | 0.19 | 0.31 - 0.44 |
| India | 8 | 5.09 | 0.12 | 0.2 - 0.28 | 0.65 | 0.48 | 0.19 | 0.32 - 0.44 |
| India | 9 | 5.12 | 0.12 | 0.2 - 0.28 | 0.65 | 0.50 | 0.20 | 0.33 - 0.46 |
| India | 10 | 4.80 | 0.12 | 0.2 - 0.28 | 0.65 | 0.43 | 0.16 | 0.27 - 0.37 |
| India | 11 | 4.74 | 0.12 | 0.2 - 0.28 | 0.65 | 0.45 | 0.17 | 0.28 - 0.39 |
| India | 12 | 4.36 | 0.12 | 0.2 - 0.28 | 0.65 | 0.49 | 0.17 | 0.28 - 0.39 |
| Japan | 9 | 3.45 | 0.12 | 0.2 - 0.28 | 0.65 | 0.30 | 0.08 | 0.14 - 0.19 |
| Japan | 10 | 3.48 | 0.12 | 0.2 - 0.28 | 0.65 | 0.34 | 0.09 | 0.15 - 0.21 |
| Japan | 11 | 3.69 | 0.12 | 0.2 - 0.28 | 0.65 | 0.40 | 0.11 | 0.19 - 0.27 |
| Japan | 12 | 3.83 | 0.12 | 0.2 - 0.28 | 0.65 | 0.41 | 0.12 | 0.21 - 0.29 |
| S Korea | 10 | 3.97 | 0.12 | 0.2 - 0.28 | 0.65 | 0.38 | 0.12 | 0.19 - 0.27 |
| S Korea | 11 | 4.00 | 0.12 | 0.2 - 0.28 | 0.65 | 0.39 | 0.12 | 0.2 - 0.28 |
| Solar irradiance (I) is calculated as the average irradiance per AEZ (see Figure S6).  f1 represents solar module efficiency. See section SM2c for assumptions on future efficiency paths.  f2 represents the average performance ratio over the lifetime of the solar infrastructure^11^.  f3 represents the land-occupation ratio, which depends partly on the sun elevation in each AEZ^44^  The average electricity output from solar energy (ρ_e_) in each AEZ is calculated using the formula: *ρ_e_ = I * f1 * f2 * f3*.  (See methods section in main article for more details, and Figure S6 for a graphical representation). | | | | | | | | |


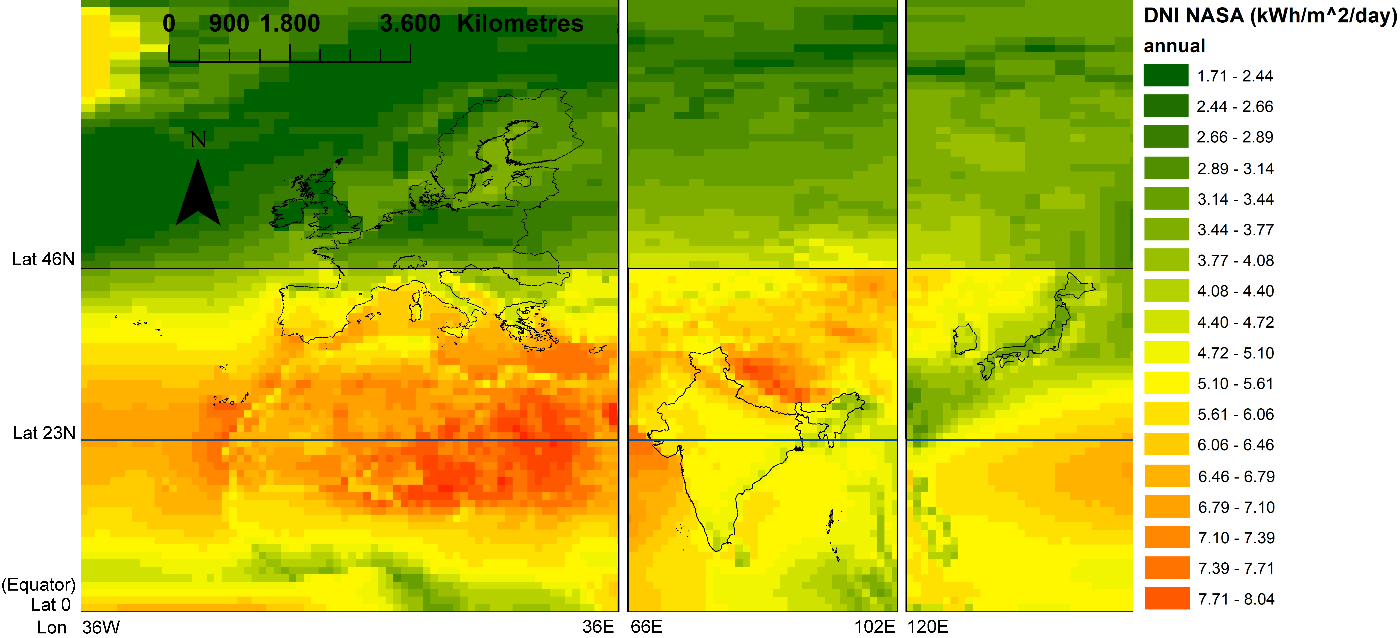


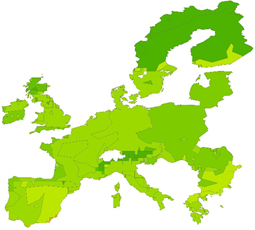

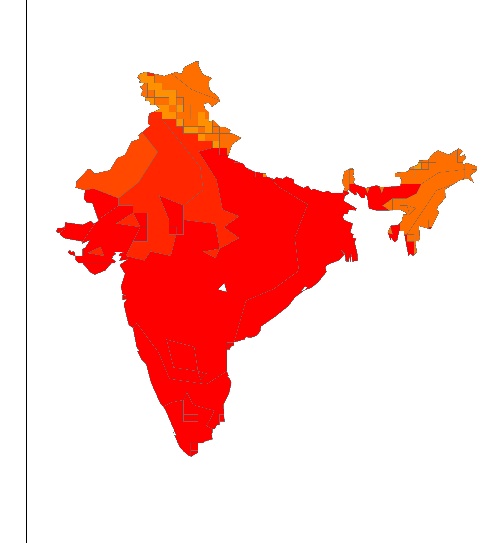

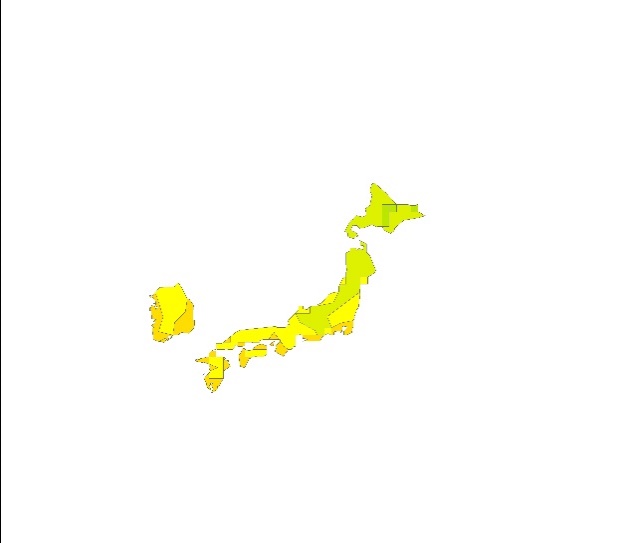

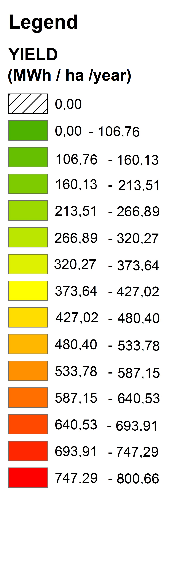


Figure S6: Above) Comparison of solar irradiance and latitude between the European Union, India, Japan and South-Korea. Source: https://power.larc.nasa.gov/ ^45^; Below) Overview of Agro-Ecological Zones solar yields calculated for each AEZ, based on the information in Table S4 (“mid efficiency” scenario, see section 2c) and illustrated in the upper part of this figure. Source: Authors´ own elaboration with the Arc GIS 10.5.1 Desktop (Esri) software.

**b) Penetration levels.** As this study focuses on future scenarios in the context of global climate action, we focus on a range of solar energy penetration scenarios that are coherent with a decarbonizing electricity mix. See Table S6 for an overview of solar energy penetration estimates in literature focusing on a largely or completely decarbonized electricity mix in 2050.

In order to cover this wide range of possible penetration rates of solar energy, we implemented scenarios aiming for a total RES (renewable electricity share; wind, solar, biomass, geothermal, hydro) ranging from 30% to 90% in 10% steps by 2050. For each penetration level in each region, there is one scenario for each technology pathway (Solar, Bioenergy and Non-land occupying renewables), where this technology becomes dominant over time. See Figure S7 for a visual representation of the modelled penetration targets for the European Union. Although Table S6 mentions predominantly scenarios with 100% RES, we did not aim for scenarios with a 100% share of RES, as backup systems based on natural gas are used at high penetration rates of intermittent energy sources (see section SM1f). Pathways for India, Japan and South-Korea were similar but starting from a different point, relevant to the actual share of RES by 2015.

Table S6: Overview of solar penetration scenarios in literature of future electricity mix

|  |  |  |  |  | **Electricity generation** | | **Share electricity generation** | |  |  |
| --- | --- | --- | --- | --- | --- | --- | --- | --- | --- | --- |
|  |  | **Target year** | **Total elect.** | **% rene-wable elect.** | **PV (incl. rooftop)** | **CSP** | **PV (incl. rooftop)** | **CSP** | **Total solar** |  |
|  | *scenario* |  | *TWh/y* | *%* | *TWh/y* | *TWh/y* | *%* | *%* | *%* | *Source* |
| ***Greenpeace 2015***^46^ | E[R] global | 2050 | 49,852 | 92% | 9,914 | 8,138 | 19.9% | 16.3% | 36.2% | Tab. 13.1.8 |
|  | ADV E[R] global | 2050 | 67,535 | 100% | 13,613 | 14,035 | 20.2% | 20.8% | 40.9% | Tab. 13.1.9 |
| ***Jacobson et al., 2017***^47^ | WWS global | 2050 | 103,368 | 100% | 49,609 | 10,081 | 48% | 9.72% | 57.6% | Tab. S6, S8 |
|  | WWS EU-28 | 2050 | 10,678 | 100% | 4,588 | 491 | 43% | 5% | 47.6% | Tab. S6, S9 |
|  | WWS India | 2050 | 8,725 | 100% | 4,248 | 1,024 | 49% | 12% | 60.4% | Tab. S6, S11 |
|  | WWS Japan | 2050 | 2,197 | 100% | 1,873 |  | 86% |  | 85.3% | Tab. S6, S10 |
|  | WWS S Korea | 2050 | 1,699 | 100% | 1,237 | 203 | 73% | 11.93% | 84.8% | Tab. S6, S10 |
| ***WWF 2011***^48^ | global | 2050 | 35,389 | 100% | 10,278 | 6,000 | 29% | 17.0% | 46.0% | Tab. A1 |
| ***LUT/EWG 2017***^49^ | global | 2050 | 48,800 | 100% |  |  | 69% | 0 | 69.0% | Figure 1 |
| ***Breyer et al., 2017***^50^ | global | 2030 | 49,408 | 100% | 21668 |  | 44% |  | 44% | Tab. 2 |
|  | Europe | 2030 | 5,127 | 100% | 1384 |  | 27% |  | 27% | Tab. 2 |
|  | India/SAARC | 2030 | 3,376 | 100% | 1880 |  | 56% |  | 56% | Tab. 2 |
|  | Northeast Asia | 2030 | 13,496 | 100% | 6986 |  | 52% |  | 52% | Tab. 2 |
| ***Bogdanov et al., 2019***^51^ | global | 2050 | 56,052 | 97.5% | 38129 |  | 68% |  | 68% | Tab S10 |
|  | Europe | 2050 | 5,931 | 94% | 2634 |  | 44.4% |  | 44.4% | Tab S12 |
|  | India/SAARC | 2050 | 8,076 | 98% | 7005 |  | 86.7% |  | 86.7% | Tab S20 |
|  | Northeast Asia | 2050 | 17,264 | 98% | 12244 |  | 70.9% |  | 70.9% | Tab S22 |

In scenarios in which bioenergy is the dominant technology, less bioenergy is used in non-electricity sectors, due to higher bioenergy prices. This leads to slightly higher mitigation costs in such scenarios, a metric that is not included in the comparison. Therefore, we can say that land use change (LUC) effects of bioenergy electricity scenarios are slightly underestimated. For scenarios with solar energy and non-land occupying technology dominance, regional bioenergy consumption is fixed. Bioenergy consumption in other regions is fixed in all scenarios, to avoid higher mitigation costs due to land pressure in other regions for scenarios with bioenergy and solar energy dominance, improving the comparability of scenario results.

Figure S7: Example of intended penetration rates of all renewables in the electricity mix of the EU (30%-90%) and dominant technology starting around 8% in 2020. The intended penetration rate of the dominant technology (either solar energy, bio-energy, or non-land occupying technologies; see methods section) slowly dominates the renewable energy mix in each scenario.

Due to various factors, such as the rigidity of the energy system (e.g. long lifespan of already installed generation capacity) and the way hydro-electricity is modelled in GCAM (as a fixed expected annual output^1^), actual penetration levels in each scenario cannot meet the intended targets. Realized penetration levels of solar energy are listed in Table S7. See section SM3a for more detailed scenario results.

Table S7: Realized penetration levels of solar energy per region

| **European Union** | 25.7 %, 34.8 %, 43.9 %, 53.0 %, 62.2 %, 71.3 %, 78.6 % |
| --- | --- |
| **India** | 30.2 %, 38.0 %, 45.9 %, 53.8 %, 61.8 %, 65.5 %, 77.8 % |
| **Japan and South Korea** | 27.5 %, 36.8 %, 46.1 %, 55.4 %, 64.8 %, 74.1 % |

**c) Future efficiency of solar energy technologies.** A wide variety of PV technologies currently exists at commercial level, and even more at research stage, with varying levels of performance. Although very high efficiencies can be obtained in research prototypes in the laboratory (>40%), the weighted average of global panel PV efficiency currently being installed (2017) is around 16%^52^. Multi-Si technology currently dominates the market with around 62% of new solar capacity (increasing trend in the last 25 years), followed by Mono-Si (33%), and the remaining 5% of capacity have been using thin-films^52^.

The efficiency of solar panels has been increasing in the last decades from ~8% in the 1980s. However, although potential for further technological improvement still exists, uncertainties exist on the future efficiency levels of PV due to factors such as thermodynamic limits (e.g. 34% for single junction cells, a.k.a Shockley–Queisser limit), increasing costs with technological complexity (e.g. multi-junction cells) and the trade-off between performance, flexibility and mineral availability^11,53–55^.

This is a key parameter in our analysis given that a higher efficiency produces the same amount of electrical power on a smaller area, i.e. reducing its land footprint. Thus, to take into account the uncertainties in future technological developments and market share, we consider three equally probable scenarios of global PV module efficiencies of capacity installed by 2050: 20, 24 and 28% (assuming a linear increase in efficiencies over time). At the lower bound (20%) of our estimations, simpler, cheaper and more flexible technologies such as thin-film (e.g. amorphous silicon or organic cells), all using more abundant minerals, would dominate the market^11,55–57^. The middle path (24%) corresponds to a scenario where multi and mono-crystalline silicon single-junction technologies dominate the market share and reach their maximum practical potential efficiency reachable at industrial production-level^58,59^. Finally, 28% reflects a scenario where more complex and expensive technologies such as multi-junction technologies or perovskite solar would take a significant share of the market by 2050^58,60^.

We have not identified efficiency paths for CSP systems separately, and therefore assume the same progress relative to current efficiencies as for PV. This efficiency parameter is set equally for all countries since the current PV market is considered to be global. It is important to take into account that, although more efficient panels might exist by 2050, it is highly unlikely that such panels will be selected for *all* investments in solar energy, as costs per unit of solar capacity are often correlated with their efficiency. Therefore, these numbers reflect the average efficiency of all newly installed solar panels by 2050.

**d) Land management regimes.** Direct LUC emissions from the conversion of other land to solarland depend on various factors, such as the previous land cover and the way solarland is managed from the point of construction onwards. Therefore, the estimation of direct LUC emissions (which can be negative if solarland is a net source of carbon sequestration), depend on some assumptions.

First of all, it is important to know the previous land cover of projected future solarland. However, while GCAM tracks total land cover by period, it does not provide specific information on what type of land changed to what other type of land (e.g. it does not project land use change in a specific area, just overall land cover by area over different periods). This means that no information can be obtained with GCAM about the type of land on which solar capacity is built. So, to estimate the previous land cover of projected future solarland, we have to analyse the obtained results in terms of land cover change on a temporal and spatial scale. Figure S8 shows land cover change over time for a representative solar expansion scenario in each of the regions, and a counterfactual scenario without expansion of USSE. The figure shows that by default (i.e. without solar expansion) in each period and region, a decline of unmanaged land uses is projected, such as grassland, scrubland and unmanaged pasture and forest, and which is driven by the increase in commercial land uses such as crop and timber production. Land competition driven by solar energy expansion is projected to, in most cases, further reduce unmanaged land uses and limit the expansion of other commercial land uses. Given that the node competition structure of the model (see Figure S2 in section SM1c) assumes an equal distance from grassland, scrubland and pasture to either forest, cropland and solarland, a part of the decline in these land covers can be assumed to be directly used for solarland, instead of cropland and forest, also since grassland, pastures, and scrublands are often suitable for solar energy. Therefore, we applied equation 5 to estimate a plausible quantity of grass- and scrubland used as solarland (*s*), analysing land cover changes over time and by AEZ. This equation assigns the decline in one type of land cover (*i;* pasture, grassland or scrubland) within an AEZ in a certain year (*t*) to all other land covers (*j*) in that AEZ that are increasing, by the proportion in which these land covers increase, and assuming a proportional share of each increasing land use. For example, if both cropland and solarland increase from 2020 to 2025, while pasture decreases, we assume that both cropland and solarland are using pasture proportional to their relative increase.

${i(s)}_{AEZ,t}=if \Delta i_{AEZ,t}<0 and \Delta s_{AEZ,t}>0; \Delta i_{AEZ,t}*\frac{-{\Delta s}_{AEZ,t}}{{\Delta s}_{AEZ,t} + \sum\Delta j_{AEZ,t} > 0};0 otherwise$ (5)

However, a different formula is applied to estimate the direct use of forest for solar energy. As we deem it less likely that forest will be converted to solarland, due to several limitations, such as physical (i.e. land suitability), economic (i.e. land preparation costs) and political (i.e. forest conservation and solar expansion are often both driven by a “green” agenda) ones. We therefore assume that the potential absolute decrease in forest, as can be observed in Figure S8, will not be directly used for solar energy as long as the increase in solarland can be assigned to the decrease in pasture, grass- and scrubland as estimated by formula 5, by the decrease or “avoided” increase of cropland compared to a scenario without solar expansion, and the observed decline in unused arable land in that period and AEZ. In other words, only if demand for solarland is so high in that there is no suitable arable land, pasture, grass- and scrubland left, forest will be directly used for hosting solar infrastructure. Figure S9 shows the estimated shares of previous land uses for projected future solarland based on the described methodology for each of the regions, and Figure S10 for one scenario by AEZ. It shows that in all three regions, mostly arable land is estimated to be used for USSE, but that pasture and grassland is also expected to host a significant share in the EU, while in Japan and South Korea, forest density is so high that up to 50% of USSE is estimated to be directly installed in cleared forest for high solar energy penetration and low solar module efficiency scenarios. These shares are not uniformly distributed over each of the three regions, and in areas with high amounts of pasture/grassland (e.g. north western Europe), scrubland (e.g. north western India), and forest (northern Japan and South Korea), higher shares of solar are estimated to be allocated such land areas. A comparison of these estimated shares in 2015 to 2020 with the observations in Table S2 is obscured through the large shares of solar power in mixed (“mosaic”) land cover. However, an attempt to translate those observations to the 4 aggregated land covers of our interest – arable land, grassland/pasture, scrubland and forest – shows a relatively good match of our 2020 estimates with the observed allocation of solar power plants (see Figure S9), partially validating the methodology applied in this study.

Note that the shares in Figure S9 are not direct outcomes from this study, but are rather estimates from which we calculate the impact of solar infrastructure on the local carbon cycle, which also depends on the land management regime. See Table S8 for all assumptions of related to the local carbon balance after a conversion to solarland. Figure S11 illustrates that in most cases, the construction of solar energy infrastructure will lead to a local net loss of carbon, though the quantity depends strongly on management decision at the point of construction and maintenance. However, if arable land is used for solar energy, and is converted to pasture by seeding the soil below the infrastructure and allowing for extensive animal grazing, there will be a net uptake of carbon locally. The amount of carbon release and uptake in each scenario depends strongly on the AEZ where the land conversion takes place, as equilibrium carbon stocks in soil differ by area and previous land use. Table S9 shows the assumed local net LUC emissions due to solar energy driven land conversion in each AEZ in the focus regions of this study, and depending on the management regime applied for solar parks in that area.


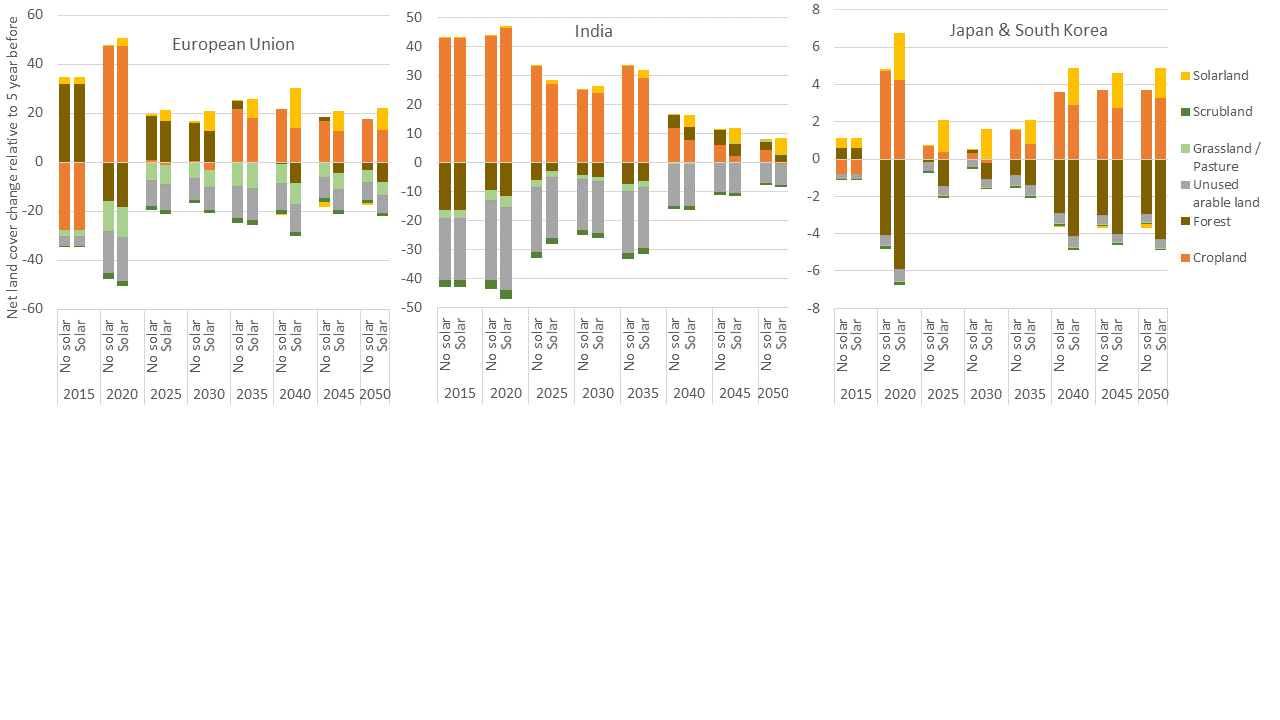


Figure S8: Projected net land cover changes by 5-year period for a scenario in which no new USSE is entering the electricity mix after 2015 (“No solar”) and a scenario where USSE will account for 45-52% of the electricity mix by 2050 (“mid scenario” for PV module efficiencies). Different penetration and efficiency levels for solar energy lead to relatively proportional results as in this example.


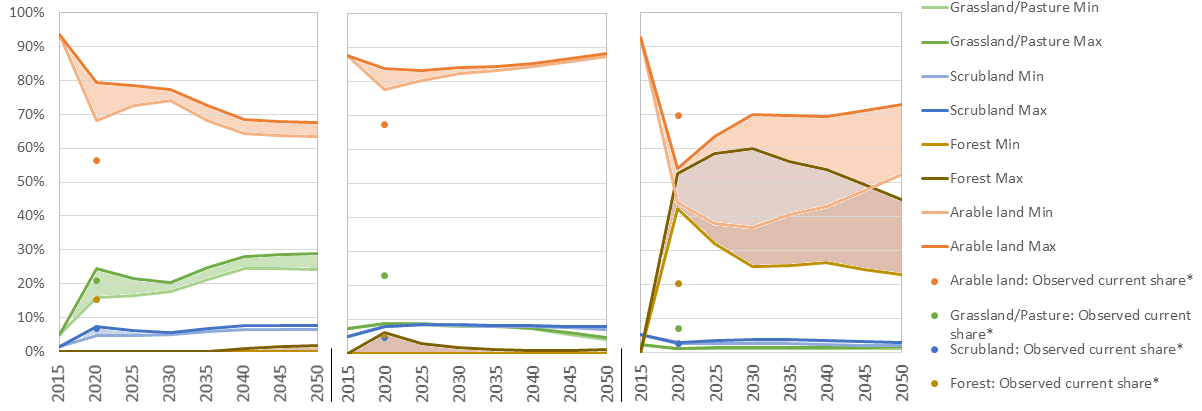


Figure S9: Estimated previous land use shares in projected future solarland for a range of solar penetration scenarios (all penetration and efficiency levels), and observed previous land use shares of current solar energy calculated from Table S2, by region. See Figure S11 for shares per AEZ within each region. *Observed current shares are based on the geolocation of 5-20% of the current capacity in each of the regions, as mentioned in Table S2, and land cover regions in Table S2 are aggregated to 4 gross types (arable land, forest, grassland/pasture and scrubland). For this aggregation, bare areas and land with sparse vegetation is treated as arable land due to its classification in GCAM, and for “mosaic” land covers, an average proportion is assumed for the 4 land covers in this figure, i.e. “Mosaic cropland (50-70%) / vegetation (grassland/shrubland/forest) (20-50%)” is treated as 60% cropland, and 11.67% grassland, 11.67% scrubland and 11.67% forest. Solar energy in “Artificial surfaces” and “Water bodies” have been excluded for this comparison.

**
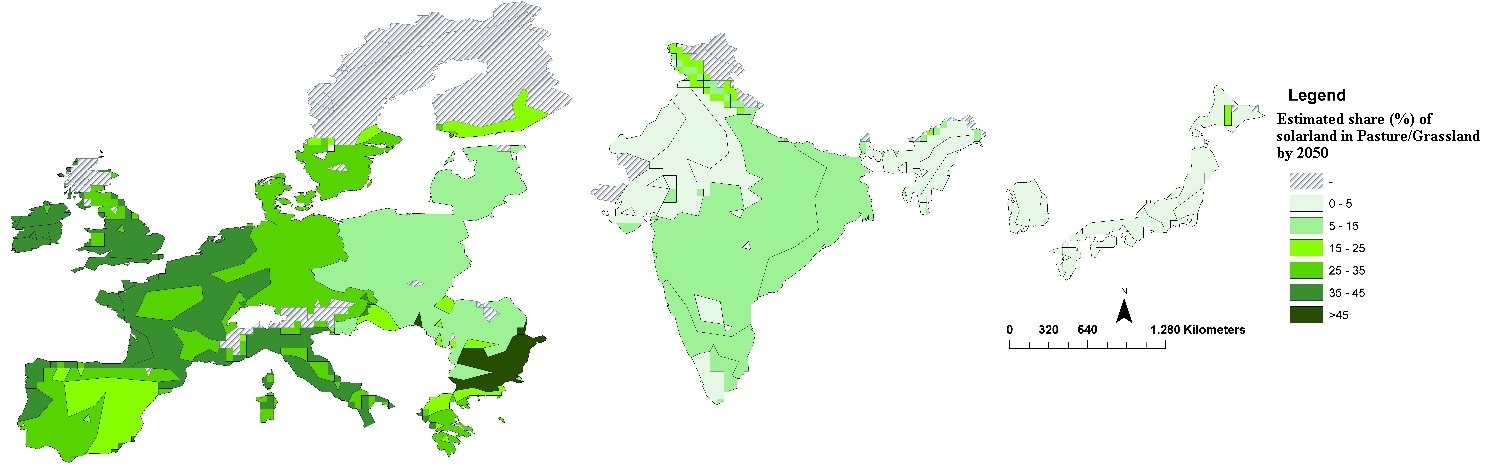

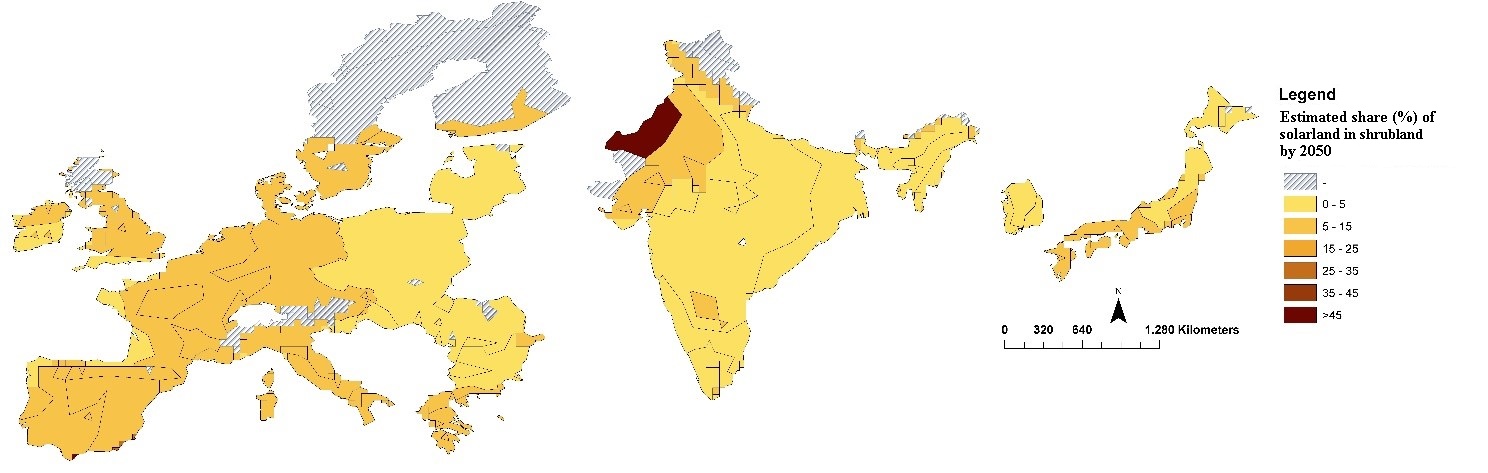
**

**
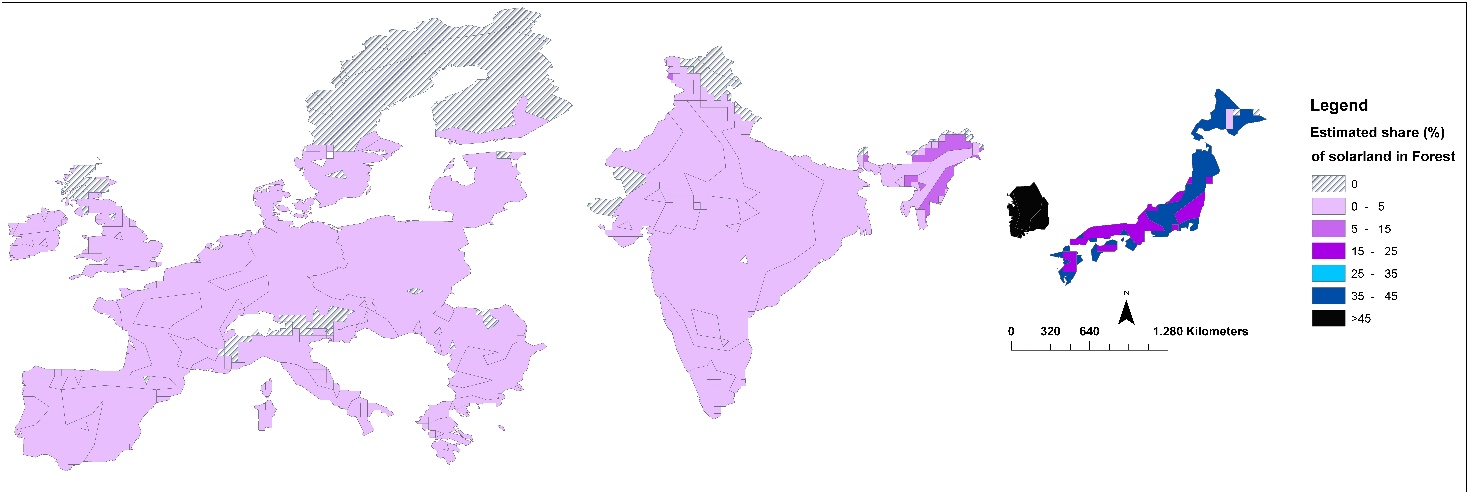
**

**
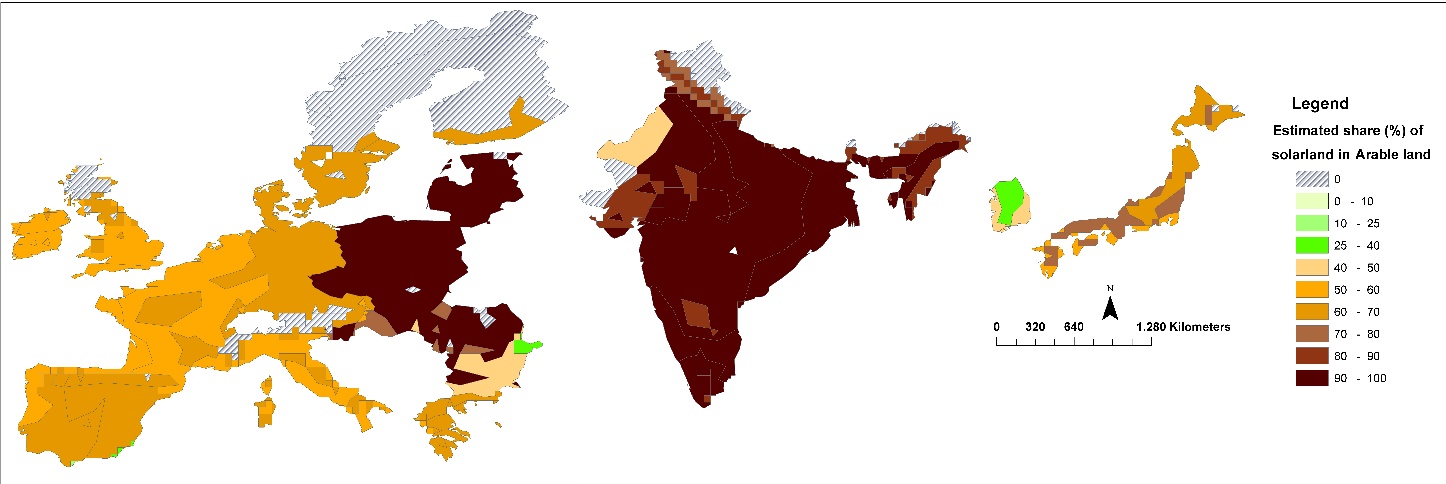
**

Figure S10: Plausible share of solarland directly installed in grassland and scrubland per region and AEZ in a scenario with 53-55% solar penetration and 24% module efficiency by 2050, estimated by equation 5. Source: Authors´ own elaboration with the Arc GIS 10.5.1 Desktop (Esri) software.

Table S8: Assumptions made for vegetation and soil carbon stocks in solarland by land management regime, depending on estimated previous land use of projected solarland (see Figure S9).

| *Previous land cover 🡪* | **Arable land** | | **Pasture** | | **Forest / natural grassland** | | **Scrubland** | |
| --- | --- | --- | --- | --- | --- | --- | --- | --- |
| *Land management regime:* | **Vegetation** | **Soil** | **Vegetation** | **Soil** | **Vegetation** | **Soil** | **Vegetation** | **Soil*** |
| **Permanently clearing land vegetation**^61,62^ | 0.1 tC/ha in “gap” area, 0.02 tC/ha in “under” area | No change | 0.1 tC/ha in “gap” area, 0.02 tC/ha in “under” area | Same as arable land | 0.1 tC/ha in “gap” area, 0.02 tC/ha in “under” area | Same as arable land | 0.1 tC/ha in “gap” area, 0.02 tC/ha in “under” area | Decreases by above-ground C removal * 0.25 (shoot-to-root ratio) |
| **Maintain/ restore previous vegetation (up to 30 cm)** | 0.2 tC/ha in “gap” area, 0.1 tC/ha in “under” area | No change | Unchanged in “gap” area, 0.1 tC/ha in “under” area | No change in “gap” area, equal to arable land in “under” areas | Equal to pasture in “gap” area, 0.1 tC/ha in “under” area | Equal to pas-ture in “gap” areas, equal to arable land in “under” areas | Equal to pasture in “gap” area, 0.1 tC/ha in “under” area | Decreases by above-ground C removal * 0.25 (shoot-to-root ratio) |
| **Seeding and management as pastures**^63,64^ | same as grassland in “gap” area, 0.18 tC/ha in “under” area | Equal to pasture level in “gap” area, no change in “under” area | Unchanged in “gap” area, 0.18 tC/ha in “under” area | No change in “gap” area, equal to arable land in “under” area | Equal to pasture in “gap” area, 0.18 tC/ha in “under” area | Equal to pas-ture in “gap” area, equal to arable land in “under” area | Equal to pasture in “gap” area, 0.18 tC/ha in “under” areas | Decreases by above-ground C removal * 0.25 (shoot-to-root ratio) |
| See Table S1 for default values of carbon stocks assumed in GCAM  tC/ha = Tons of Carbon per hectare  “under” area can be interpreted as area below solar panels/heliostats (f3 value in equation 4 in Methods section of main article)  “gap” area can be interpreted as land between rows of panels/heliostats that is not covered (1 – f3)  Values for 0.1 and 0.2 tC/ha for “gap” areas are based on whether or not spontaneous vegetation (e.g. weeds) is limited by applying herbicides.  Values of 0.02, 0.1 and 0.18 tC/ha for “under” areas are based on management regime narratives:   - 0.02 tC/ha is based on virtually no vegetation in “under” areas, due to application of herbicides and low placement of panels, representing zero interest in carbon sequestration in solarland. - 0.1 tC/ha is based on no application of herbicides, and slightly higher placed panels to avoid shadow from vegetation (up to 30 cm) in “gap” areas. - 0.18 tC/ha is based on higher placed panels to allow some sunlight and small grazers to reach the “under” areas, as well as herb seeding of these areas (which are still in the shadow most of the time).   *We only used a “root-to-shoot” ratio for estimating impacts on scrubland soil, because default GCAM estimates for scrubland are not very elaborated. Using this ratio, a good approximation can be done about the impacts of vegetation removal on soil carbon stocks | | | | | | | | |


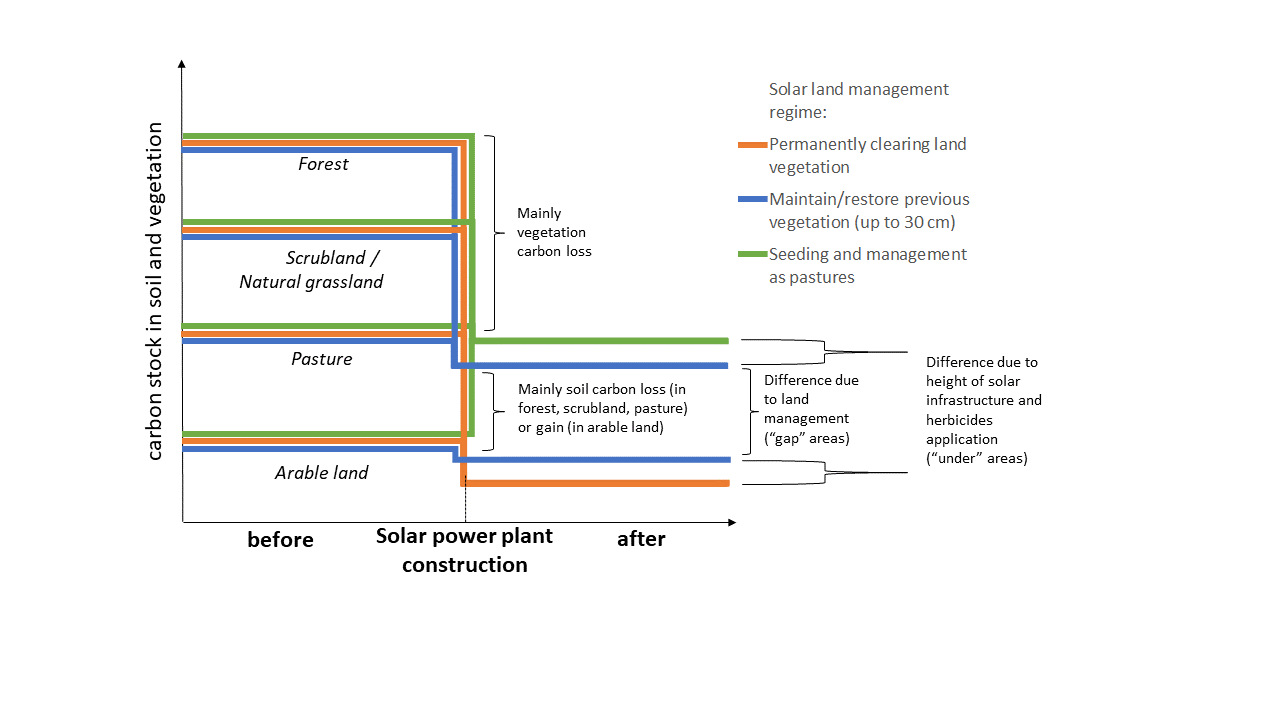


Figure S11: Graphical representation of the impact of solarland conversion on carbon stocks in land, by previous land cover and by land management regime.

| Table S9: Values of carbon release/uptake in vegetation and soil due to conversion to solarland, depending on region, AEZ, and estimated previous land cover (see Figure S9)  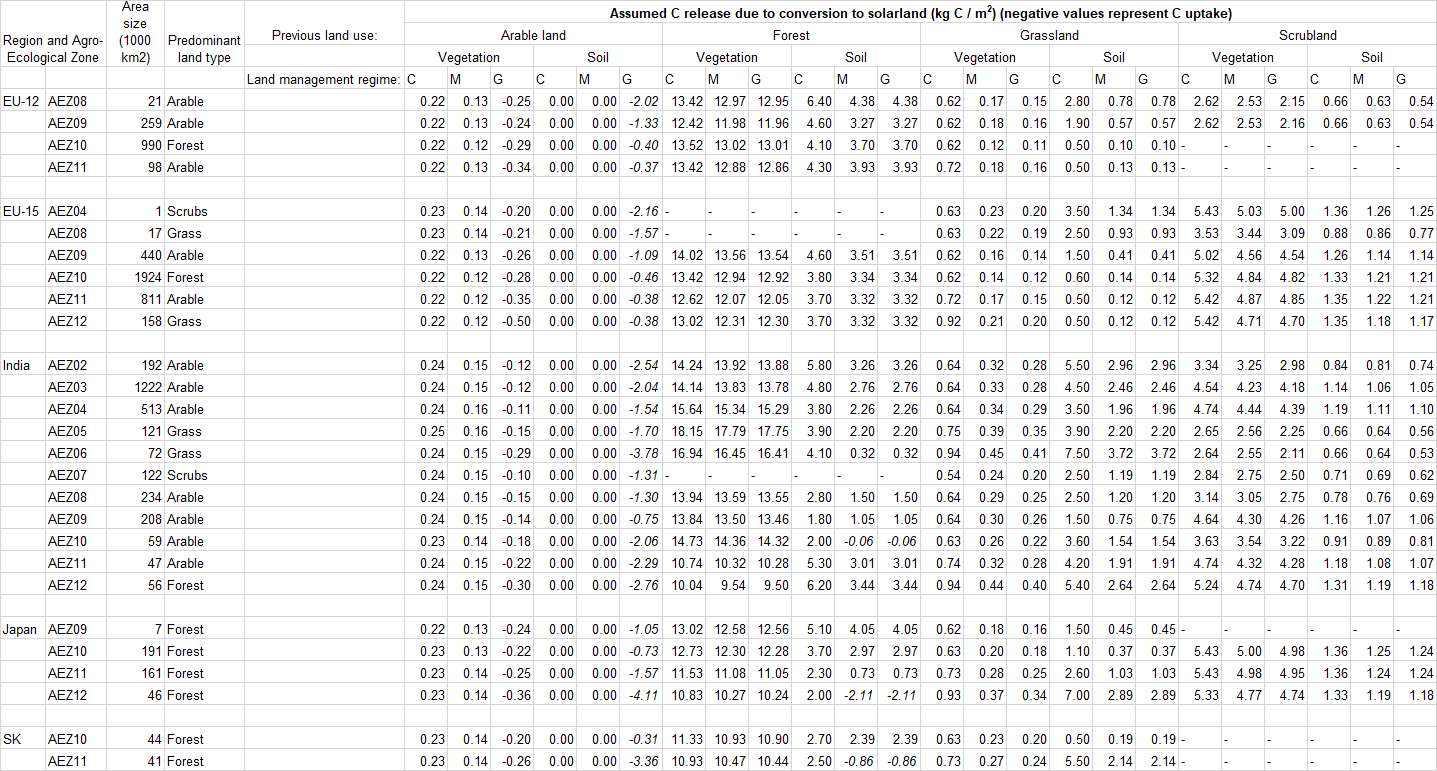 |
| --- |
| **C = Permanently clearing land vegetation**  **M = Maintain/ restore previous vegetation (up to 30 cm)**  **G = Seeding and management as pastures**  Vegetation C release occurs instantly, soil C release through an asymptotic function (most in the first years, less thereafter)  Vegetation C uptake occurs through a Bertalanffy-Richards function^2^, soil C uptake (for pasture conversion, see values in *italics*) by constant rate of 0.35 tC / hectare / year^65,66^ |

**Section 3: Scenario outcomes**

**a) Solar penetration scenarios.** All results in this study have been reported by their solar penetration level reached in 2050. To give some interpretation of how the modelled scenarios evolved from the calibrated base year (2015) until 2050, Figure S12 shows the solar output and total electricity output per year in all modelled scenarios. While final electricity demand is fixed in all scenarios, total electricity production increases with higher solar penetration due to the increasing needs for gas-powered back-up capacity (see Section SM2f).

Figure S13 shows how the technological breakdown of the installed solar energy capacity by 2050 in each scenario. In all regions, the majority of solar energy comes from land-based utility-scale PV, in some cases with on-site storage systems and about 12% of Indian capacity is installed in identified “wastelands”. Residential PV by 2050 in each region does hardly increase with increasing penetration levels, which suggests that all urban space with acceptable solar energy efficiency will be used by 2050 in each of the scenarios.


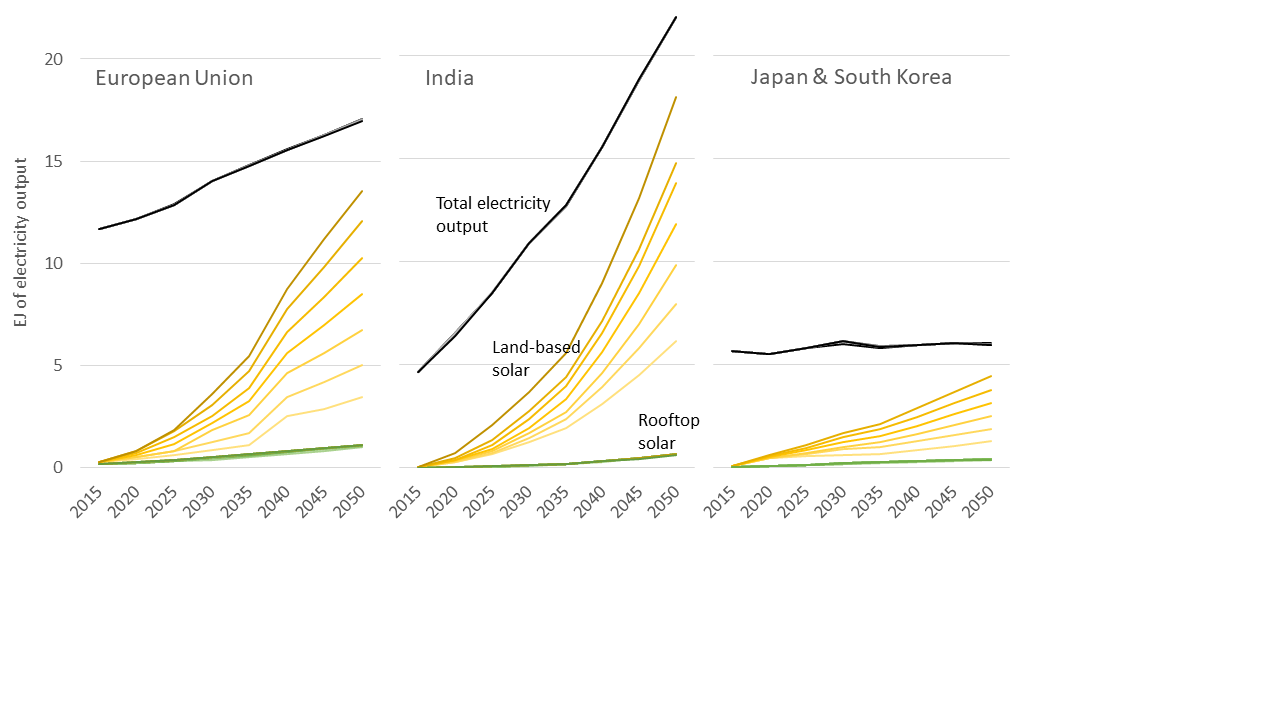


Figure S12: Solar penetration over time for each scenario. Darker lines represent higher solar penetration scenarios. See Section SM1b for more details on the modelled penetration levels.


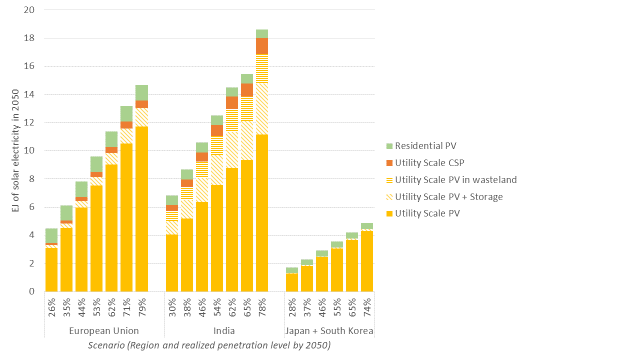


Figure S13: Solar electricity in 2050 by technology for each scenario (reaching 24% average PV module efficiency in 2050)

**b) Land cover changes.** Regional and “leaking” land cover changes as reported in Figure 2 in the main text, add up to net total land cover changes on a global level, shown in Figure S14 This figure shows more clearly how high penetration levels in the EU, Japan and South Korea lead to a net increase in global cropland cover, while global commercial forest cover shows a net increase due to solar energy installed in India.


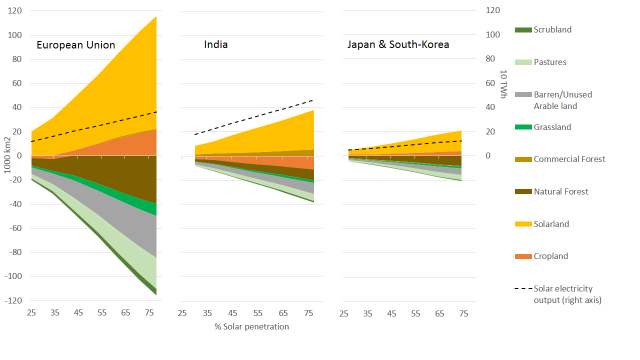


Figure S14: Total net global land cover changes by 2050 by type for each region (for scenarios reaching a 24% average PV module efficiency by 2050). See Figure 2 in main document for relative changes within each of the regions, and outside the regions.

**c) CO_2_ payback period.** To support the results in terms of CO_2_ payback periods for solar- and bio-energy, Figure S15 shows these differences in the payback period in a more illustrative way for a single penetration and efficiency scenario. The horizontal lines represent the total amount of LUC emissions due to land cover changes in each scenario. This figure does only focus at solar and bio-energy based in land suitable for commercial purposes. Solar energy in urban areas, deserts and dry scrublands, as well as bio-energy from waste or agricultural residue, is assumed not to contribute to any LUC emissions. The CO_2_ payback per period is linked to the LUC emission inducing share of installed solar and bioenergy. Since this part is relatively smaller in bioenergy compared to solar energy scenarios (e.g. more bioenergy from waste streams than solar energy from rooftops), CO_2_ payback per period is smaller for bioenergy scenarios in all regions.


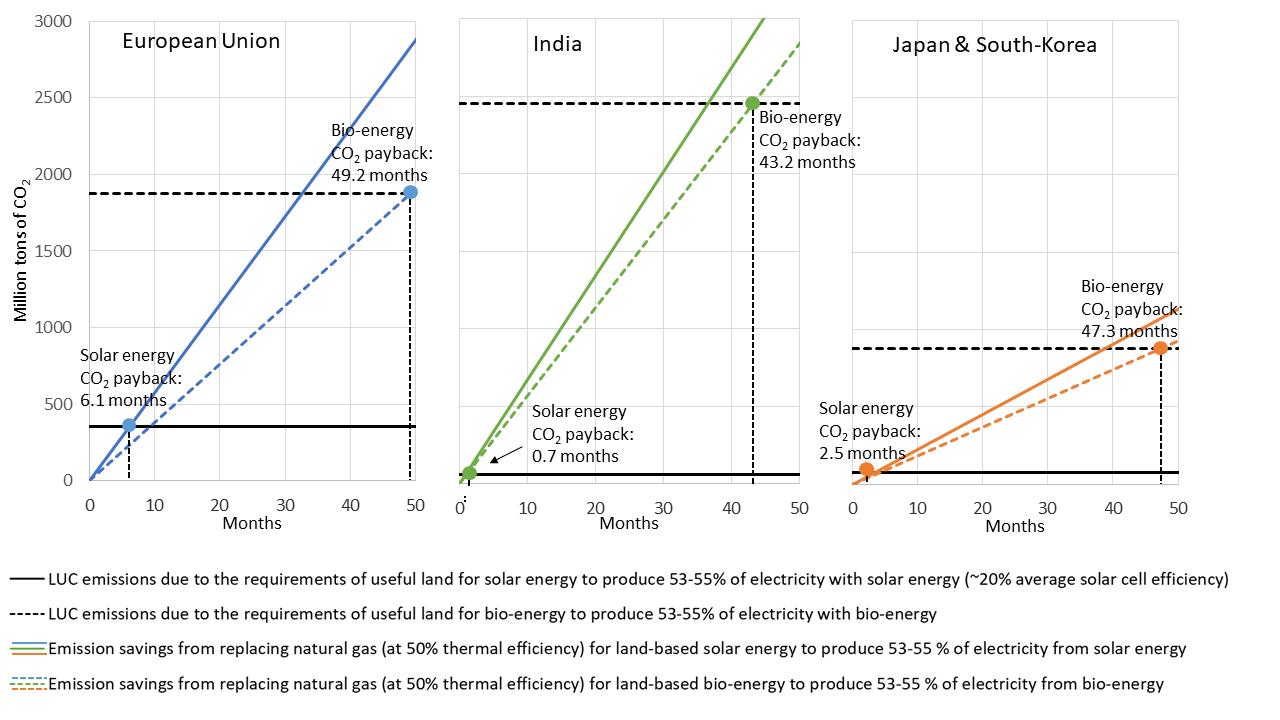


Figure S15: Graphical representation of CO_2_ pay-off principle, showing scale differences between solar- and bio-energy and between regions. For 53-55% penetration scenarios and solar module efficiency ranging from 16% in 2020 to 24% in 2050, and “Maintain” land management regime.

**d) Solarland as % of agricultural land by AEZ**

**
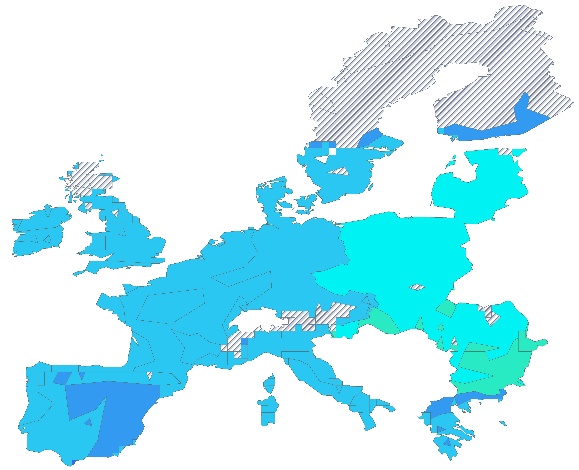

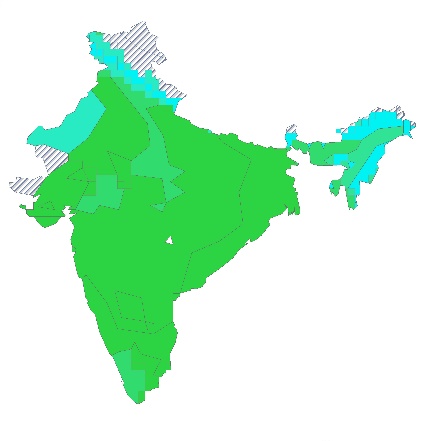

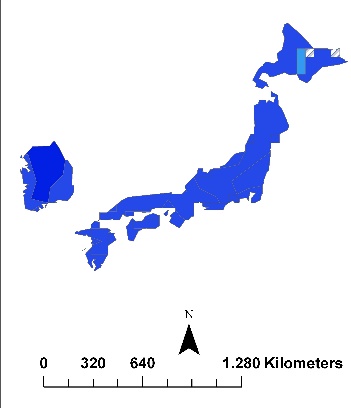

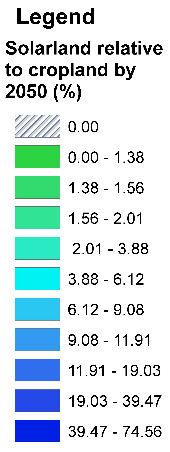
**

Figure S16: Relative amount of solarland compared to cropland in each region and AEZ for ¬54% penetration and 24% module efficiency by 2050. Source: Authors´ own elaboration with the Arc GIS 10.5.1 Desktop (Esri) software.

**References**

1. JGCRI. GCAM v4.3 Documentation. http://jgcri.github.io/gcam-doc/v4.3/toc.html (2016).

2. Lei, Y. C. & Zhang, S. Y. Features and partial derivatives of Bertalanffy-Richards growth model in forestry. *Nonlinear Anal. Model. Control* **9**, 65–73 (2004).

3. McFadden, D. Conditional logit analysis of qualitative choice behavior. in *Frontiers in Econometrics* (Academic Press, 1974).

4. WISE, M., CALVIN, K., KYLE, P., LUCKOW, P. & EDMONDS, J. ECONOMIC AND PHYSICAL MODELING OF LAND USE IN GCAM 3.0 AND AN APPLICATION TO AGRICULTURAL PRODUCTIVITY, LAND, AND TERRESTRIAL CARBON. *Clim. Chang. Econ.* **05**, 1450003 (2014).

5. Kyle, G. P. *et al.* *GCAM 3.0 Agriculture and Land Use: Data Sources and Methods*. http://www.osti.gov/servlets/purl/1036082/ (2011) doi:10.2172/1036082.

6. Houghton, R. A. The annual net flux of carbon to the atmosphere from changes in land use 1850–1990*. *Tellus B* **51**, 298–313 (1999).

7. King, A. W., Post, W. M. & Wullschleger, S. D. The Potential Response of Terrestrial Carbon Storage to Changes in Climate and Atmospheric CO2. *Clim. Change* **35**, 199–227 (1997).

8. Clarke, J. F. & Edmonds, J. A. Modelling energy technologies in a competitive market. *Energy Econ.* **15**, 123–129 (1993).

9. Train, K. *Discrete choice methods with simulation*. (Cambridge university press, 2003).

10. ATLAS. *Wasteland atlas of India*. http://www.dolr.nic.in/WastelandsAtlas2011/Wastelands_Atlas_2011.pdf (2011).

11. De Castro, C., Mediavilla, M., Miguel, L. J. & Frechoso, F. Global solar electric potential: A review of their technical and sustainable limits. *Renew. Sustain. Energy Rev.* **28**, 824–835 (2013).

12. De Marco, A. *et al.* The contribution of Utility-Scale Solar Energy to the global climate regulation and its effects on local ecosystem services. *Glob. Ecol. Conserv.* **2**, 324–337 (2014).

13. Dias, L., Gouveia, J. P., Lourenço, P. & Seixas, J. Interplay between the potential of photovoltaic systems and agricultural land use. *Land use policy* **81**, 725–735 (2019).

14. Vrînceanu, A. *et al.* Impacts of Photovoltaic Farms on the Environment in the Romanian Plain. *Energies* **12**, 2533 (2019).

15. Adeh, E. H., Good, S. P., Calaf, M. & Higgins, C. W. Solar PV Power Potential is Greatest Over Croplands. *Sci. Rep.* **9**, 11442 (2019).

16. Hernandez, R. R. *et al.* Solar energy development impacts on land cover change and protected areas. *Proc. Natl. Acad. Sci.* **113**, E1768–E1768 (2016).

17. Van Den Pol-Van Dasselaar, A. *et al.* Beweidbare oppervlakte en weidegang op melkveebedrijven in Nederland. *Wageningen UR (University Res. centre) Livest. Res. Rapp.* **917**, (2015).

18. IEA. *2018 SNAPSHOT OF GLOBAL PHOTOVOLTAIC MARKETS*. (2018).

19. Mahtta, R., Joshi, P. K. & Jindal, A. K. Solar power potential mapping in India using remote sensing inputs and environmental parameters. *Renew. Energy* **71**, 255–262 (2014).

20. Deng, Y. Y. *et al.* Quantifying a realistic, worldwide wind and solar electricity supply. *Glob. Environ. Chang.* **31**, 239–252 (2015).

21. de Vries, B. J. M., van Vuuren, D. P. & Hoogwijk, M. M. Renewable energy sources: Their global potential for the first-half of the 21st century at a global level: An integrated approach. *Energy Policy* **35**, 2590–2610 (2007).

22. de Castro, C. & Capellán-Pérez, I. Concentrated Solar Power: Actual Performance and Foreseeable Future in High Penetration Scenarios of Renewable Energies. *Biophys. Econ. Resour. Qual.* **3**, 14 (2018).

23. IEA. *Energy Balances of OECD Countries 1960-2015 and Energy Balances of Non-OECD Countries 1971-2015*. (International Energy Agency, 2017).

24. UNFCCC. UNFCCC, NDC Registry. https://www4.unfccc.int/sites/NDCStaging/Pages/All.aspx (2019).

25. SolarPower Europe. *Global Market Outlook for Solar Power 2015-2019*. *Global Market Outlook* (2014) doi:10.1787/key_energ_stat-2014-en.

26. Hahn, E. *The Japanese Solar PV Market and Industry*. *Minerva Fellowship Programme* https://www.eu-japan.eu/publications/japanese-solar-pv-market-and-industry-business-opportunities-european-companies (2014).

27. IEA PVPS. *Trends in Photovoltaic Applications 2019*. (2019).

28. IEA PVPS. *Snapshot of Global PV Markets 2020*. (2020).

29. Capellán-Pérez, I., de Castro, C. & Arto, I. Assessing vulnerabilities and limits in the transition to renewable energies: Land requirements under 100% solar energy scenarios. *Renew. Sustain. Energy Rev.* **77**, 760–782 (2017).

30. Muratori, M. *et al.* Cost of power or power of cost: A U.S. modeling perspective. *Renew. Sustain. Energy Rev.* **77**, 861–874 (2017).

31. IEA. Executive summary: Projected Costs of Generating Electricity. (2015).

32. Breyer, C. *et al.* North-East Asian super grid: renewable energy mix and economics. *Jpn. J. Appl. Phys.* **54**, 08KJ01 (2015).

33. DoE, U. S. *Concentrating solar power commercial application study: reducing water consumption of concentrating solar power electricity generation*. *Report to Congress. Washington, DC: USDOE* (2009).

34. Farrell, J. *Is Bigger Best in Renewable Energy?* https://ilsr.org/report-is-bigger-best/ (2016).

35. McKinsey & Company. *Transformation of Europe’s power system until 2050 Including specific considerations for Germany Electric Power and Natural Gas Practice*. https://www.mckinsey.com/~/media/mckinsey/dotcom/client_service/epng/pdfs/transformation_of_europes_power_system.ashx (2010).

36. Wise, M., Kyle, P., Luckow, P. & Clarke, L. Representing Wind and Solar Energy in GCAM: Current Issues. in (2010).

37. Denholm, P. & Margolis, R. M. Land-use requirements and the per-capita solar footprint for photovoltaic generation in the United States. *Energy Policy* **36**, 3531–3543 (2008).

38. Hamelinck, C. N., Suurs, R. A. A. & Faaij, A. P. C. International bioenergy transport costs and energy balance. *Biomass and Bioenergy* **29**, 114–134 (2005).

39. Klein Goldewijk, K., Beusen, A., van Drecht, G. & de Vos, M. The HYDE 3.1 spatially explicit database of human-induced global land-use change over the past 12,000 years. *Glob. Ecol. Biogeogr.* **20**, 73–86 (2011).

40. IEA. *Energy Balances of OECD Countries 1960-2010 and Energy Balances of Non-OECD Countries 1971-2010*. (International Energy Agency, 2012).

41. E3MLab & IIASA. *Technical report on Member State results of the EUCO policy scenarios*. https://ec.europa.eu/energy/sites/ener/files/documents/20161219_-_technical_report_on_euco_scenarios_primes.pdf (2016) doi:10.1109/BEC.2012.6376833.

42. Lennon, A. South Korea targets 58.5GW of renewables by 2030. *PV Tech* https://www.pv-tech.org/news/south-korea-announce-8th-basic-plan-for-long-term-energy-generation (2017).

43. O’Neill, B. C. *et al.* A new scenario framework for climate change research: the concept of shared socioeconomic pathways. *Clim. Change* **122**, 387–400 (2014).

44. Martín-Chivelet, N. Photovoltaic potential and land-use estimation methodology. *Energy* **94**, 233–242 (2016).

45. NASA Langley Atmospheric Sciences Data Center. Average Monthly and Annual Direct Normal Irradiance Data, One-Degree Resolution of the World, 1983-2005. https://geo.nyu.edu/catalog/stanford-fd535zg0917.

46. Greenpeace. Energy [R]evolution 2015. (2015).

47. Jacobson, M. Z. *et al.* 100% Clean and Renewable Wind, Water, and Sunlight All-Sector Energy Roadmaps for 139 Countries of the World. *Joule* **1**, 108–121 (2017).

48. Singer, S. *et al.* *WWF ENERGY REPORT: 100% RENEWABLE ENERGY BY 2050*. (2011).

49. Pam, M. *et al.* Global energy system based on 100% renewable energy - power sector. 143 (2017).

50. Breyer, C. *et al.* On the role of solar photovoltaics in global energy transition scenarios. *Prog. Photovoltaics Res. Appl.* **25**, 727–745 (2017).

51. Bogdanov, D. *et al.* Radical transformation pathway towards sustainable electricity via evolutionary steps. *Nat. Commun.* **10**, 1–16 (2019).

52. Fraunhofer Institute for Solar Energy Systems. Photovoltaics Report. in (2018).

53. Grandell, L. & Höök, M. Assessing rare metal availability challenges for solar energy technologies. *Sustain.* **7**, 11818–11837 (2015).

54. Valero, A., Valero, A., Calvo, G. & Ortego, A. Material bottlenecks in the future development of green technologies. *Renew. Sustain. Energy Rev.* **93**, 178–200 (2018).

55. Nathan S. Lewis. Research opportunities to advance solar energy utilization. *Science (80-. ).* **351**, aad1920-1–9 (2016).

56. Kaltenbrunner, M. *et al.* Ultrathin and lightweight organic solar cells with high flexibility. *Nat. Commun.* **3**, 770 (2012).

57. Shukla, A. K., Sudhakar, K. & Baredar, P. A comprehensive review on design of building integrated photovoltaic system. *Energy Build.* **128**, 99–110 (2016).

58. Mayer, J. N., Philipps, D. S., Hussein, N. S., Schlegl, D. T. & Senkpiel, C. *Current and Future Cost of Photovoltaics*. https://www.ise.fraunhofer.de/en/publications/studies/studie-current-and-future-cost-of-photovoltaics-long-term-scenarios-for-market-development-system-prices-and-lcoe-of-utility-scale-pv-systems.html (2015).

59. Swanson, R. M. Approaching the 29% limit efficiency of silicon solar cells. in *Conference Record of the Thirty-first IEEE Photovoltaic Specialists Conference, 2005.* 889–894 (2005). doi:10.1109/PVSC.2005.1488274.

60. Philipps, S. P., Bett, A. W., Nozik, A. J., Conibeer, G. & Beard, M. C. III–V multi-junction solar cells. *Adv. Concepts Photovoltaics* **11**, 87–117 (2014).

61. NREL. *Native Vegetation Performance under a Solar PV Array at the National Wind Technology Center*. (2017) doi:NREL/TP-1900-66218.

62. MITECO. *ESTUDIO AMBIENTAL ESTRATÉGICO PLAN NACIONAL INTEGRADO DE ENERGÍA Y CLIMA 2021-2030*. https://energia.gob.es/es-es/Participacion/Paginas/DetalleParticipacionPublica.aspx?k=236 (2020).

63. Armstrong, A., Ostle, N. J. & Whitaker, J. Solar park microclimate and vegetation management effects on grassland carbon cycling. *Environ. Res. Lett.* **11**, 74016 (2016).

64. Adeh, E. H., Selker, J. S. & Higgins, C. W. Remarkable agrivoltaic influence on soil moisture, micrometeorology and water-use efficiency. *PLoS One* **13**, e0203256 (2018).

65. Qin, Z., Dunn, J. B., Kwon, H., Mueller, S. & Wander, M. M. Soil carbon sequestration and land use change associated with biofuel production: Empirical evidence. *GCB Bioenergy* **8**, 66–80 (2016).

66. Post, M. & Kwon, K. C. Soil Carbon Sequestration and Land-Use Change : Processes and Potential. (2000).
